# Bayesian Phylogenetic Inference using Relaxed-clocks and the Multispecies Coalescent

**DOI:** 10.1093/molbev/msac161

**Published:** 2022-07-30

**Authors:** Tomáš Flouri, Jun Huang, Xiyun Jiao, Paschalia Kapli, Bruce Rannala, Ziheng Yang

**Affiliations:** Department of Genetics, Evolution, and Environment, University College London, Gower Street, London WC1E 6BT, UK; Department of Genetics, Evolution, and Environment, University College London, Gower Street, London WC1E 6BT, UK; School of Biomedical Engineering, Capital Medical University, Beijing 100069, China; Department of Genetics, Evolution, and Environment, University College London, Gower Street, London WC1E 6BT, UK; Department of Statistics and Data Science, China Southern University of Science and Technology, Shenzhen, Guangdong 518055, China; Department of Genetics, Evolution, and Environment, University College London, Gower Street, London WC1E 6BT, UK; Department of Evolution and Ecology, University of California, Davis, CA 95616, USA; Department of Genetics, Evolution, and Environment, University College London, Gower Street, London WC1E 6BT, UK

**Keywords:** multispecies coalescent, molecular clock, relaxed clock, bpp, species tree

## Abstract

The multispecies coalescent (MSC) model accommodates both species divergences and within-species coalescent and provides a natural framework for phylogenetic analysis of genomic data when the gene trees vary across the genome. The MSC model implemented in the program bpp assumes a molecular clock and the Jukes–Cantor model, and is suitable for analyzing genomic data from closely related species. Here we extend our implementation to more general substitution models and relaxed clocks to allow the rate to vary among species. The MSC-with-relaxed-clock model allows the estimation of species divergence times and ancestral population sizes using genomic sequences sampled from contemporary species when the strict clock assumption is violated, and provides a simulation framework for evaluating species tree estimation methods. We conducted simulations and analyzed two real datasets to evaluate the utility of the new models. We confirm that the clock-JC model is adequate for inference of shallow trees with closely related species, but it is important to account for clock violation for distant species. Our simulation suggests that there is valuable phylogenetic information in the gene-tree branch lengths even if the molecular clock assumption is seriously violated, and the relaxed-clock models implemented in bpp are able to extract such information. Our Markov chain Monte Carlo algorithms suffer from mixing problems when used for species tree estimation under the relaxed clock and we discuss possible improvements. We conclude that the new models are currently most effective for estimating population parameters such as species divergence times when the species tree is fixed.

## Introduction

The multispecies coalescent (MSC) model (Rannala and Yang 2003) combines the phylogenetic process of species divergence with the population genetic process of coalescent, providing a framework for phylogenetic analysis of population samples (single or multi-individual) of genomic sequence data from multiple species. The MSC naturally accommodates gene tree fluctuations across the genome and potential gene-tree vs. species-tree discordance caused by incomplete lineage sorting (ILS). ILS can occur when gene sequences from different species coalesce not in their most recent common ancestral species but in an older ancestor (Maddison 1997; Nichols 2001; Szollosi et al. 2015). MSC-based methods have proven useful for resolving challenging species phylogenies with short branches that arose from a rapid succession of speciation events (Edwards et al. 2016; Xu and Yang 2016). See Edwards (2009), Rannala et al. (2020) and Jiao et al. (2021) for recent reviews of the MSC and its use in species tree estimation.

Full-likelihood (maximum likelihood or ML and Bayesian) methods of inference under the MSC applied to multilocus sequence alignments average over the gene tree topologies and coalescent times (node ages in gene trees) underlying the data at each locus (Rannala and Yang 2003; Burgess and Yang 2008; Yang and Rannala 2014; Ogilvie et al. 2016; Rannala and Yang 2017; Douglas et al. 2022). The methods make full use of information in the gene trees, whereas accommodating their uncertainties. Although such methods are computationally far more demanding than heuristic methods using summary statistics, recent breakthroughs in MCMC proposal algorithms, especially those that make coordinated changes to the species tree and the gene trees at all loci (Rannala and Yang 2003, 2013; Yang and Rannala 2014; Jones 2017; Rannala and Yang 2017; Douglas et al. 2022), have improved the mixing efficiency considerably. As a result, the Bayesian MSC program bpp has been successfully applied to genome-scale datasets with more than 10,000 loci, at least for a small number of species or sequences per locus (Shi and Yang 2018; Thawornwattana et al. 2018, 2022).

A limitation of the current MSC implementation in bpp is that it assumes a strict molecular clock and the simple Jukes–Cantor (JC; Jukes and Cantor 1969) model of nucleotide substitution, making it best suited for use with data from closely related species. For such species, sequences are highly similar and a strict clock may be approximately correct, whereas the JC model may be adequate for accounting for multiple substitutions at the same site. For distantly related species, however, the molecular clock may be seriously violated, and the JC model may be too simplistic for multiple-hit corrections. It is important to note that ILS or coalescent is as relevant to deep phylogenies as it is to shallow trees: the issue has to do with the *length* rather than *depth* of internal branches in the species tree (Edwards et al. 2005).

Over the past two decades, a number of relaxed-clock models have been developed for dating divergence events on phylogenies, allowing the substitution rate to change over time and among branches of the phylogeny; see Yang (2014, Chapter 10) and Ho (2022) for comprehensive reviews. Thorne et al. (1998) and Kishino et al. (2001) developed the earliest models, using geometric Brownian motion (GBM) to describe the evolution in the rate of molecular evolution; in other words, the logarithm of the rate drifts over time like Brownian motion. Evidence from the fossil recorded is incorporated as bounds on node ages to calibrate the tree. “Soft bounds” and arbitrary fossil-calibration densities were implemented by Yang and Rannala (2006) and Drummond et al. (2006). The independent-rates model is implemented by Drummond et al. (2006) and Rannala and Yang (2007) (see also Lepage et al. 2007), which describes the variation in rate among lineages empirically without a mechanistic basis like the GBM. Later developments include the use of dated fossils and joint analysis of morphological characters and molecular alignments in the so-called tip-dating or total-evidence dating analyses (e.g., Ronquist et al. 2012; Heath et al. 2014; Zhang et al. 2016; Alvarez-Carretero et al. 2019). See dos Reis et al. (2016), Lee and Ho (2016) and Ho (2022) for recent reviews.

**Fig. 1.**
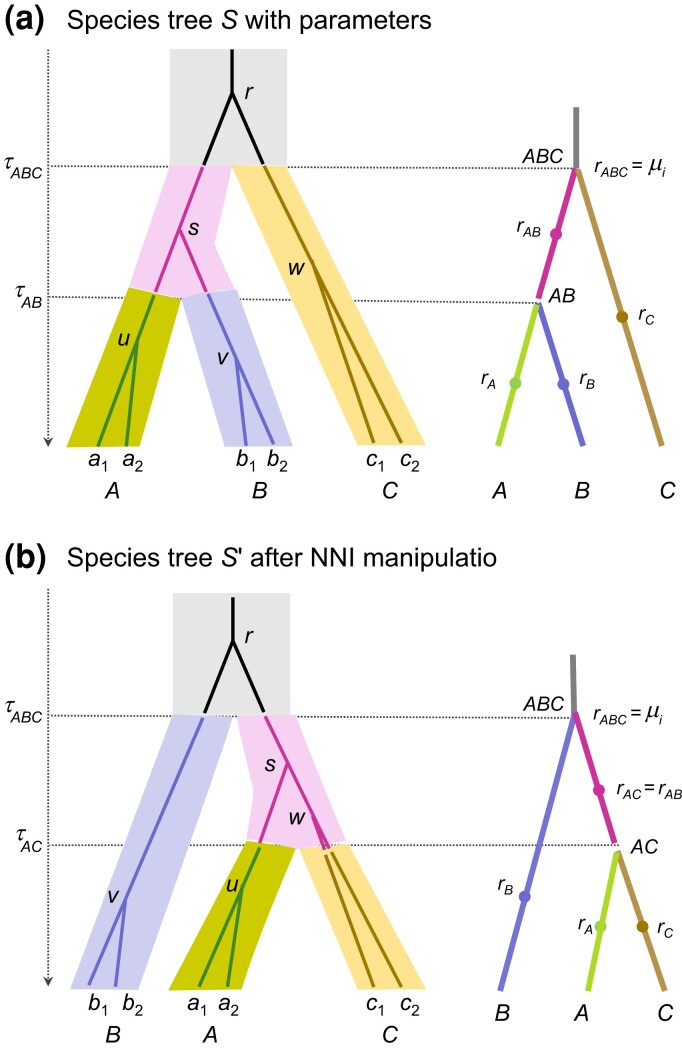
(*a*) A species tree (S) for three species (A,B,C) with a gene tree for six sequences ($a_{1},a_{2},b_{1},b_{2},c_{1},c_{2}$) inside to illustrate the parameters in the MSC+relaxed clock model. At any locus each population on the species tree has its own rate so that rates are assigned to species-tree branches, indicated by different colors. A branch on the gene tree may pass several populations, consisting of segments with different rates, and the branch length is the sum of the segments. For instance, branch su in the gene tree consists of two segments with rates $r_{A}$ and $r_{AB}$, and has the length $(\tau_{AB}-t_{u})r_{A}+(t_{s}-\tau_{AB})r_{AB}$. (*b*) Another species tree $S^{\prime }$ after an NNI/SPR perturbation of S, illustrating the mapping of branch rates at an example locus. In the NNI/SPR move under the MSC+clock model (Yang and Rannala 2014; Rannala and Yang 2017), MSC parameters (τ and θ) as well as the ages of ‘affected nodes’ on the gene trees ($t_{s}$) are transferred from S to $S^{\prime }$ without modification. For example, $\tau_{AB}$ in S becomes $\tau_{AC}$ in $S^{\prime }$, $\theta_{AB}$ in S becomes $\theta_{AC}$ in $S^{\prime }$, and $t_{s}$ in the gene tree becomes $t_{s}$ in the new gene tree. Here in the MSC+relaxed clock model, rates for (species-tree) branches at each locus are mapped onto the new species tree without modification as well (for example, $r_{AB}$ in S becomes $r_{AC}$ in $S^{\prime }$ for the locus).

In this paper, we implement relaxed-clock models in the MSC framework. We explicitly model the process of the evolution in the evolutionary rate among species, treating evolutionary rates and sequence divergence times as latent variables and averaging over them in the MCMC algorithm. We note two major differences between the MSC-relaxed clock models and the traditional phylogenetic relaxed-clock models. First, under the MSC, different genes or genomic regions may have different gene-tree topologies and coalescent times, with their distribution specified by the MSC model (Rannala and Yang 2003). In contrast, relaxed-clock methods used in phylogenetic dating do not accommodate genealogical fluctuations among genes and assume that all gene trees share a common topology, leading to potentially biased divergence time estimates (dos Reis et al. 2016; Ogilvie et al. 2016). Second, in the MSC-relaxed clock models, the rates are assigned to branches on the species tree (which represent different species), rather than branches on the gene tree (Xu and Yang 2016; Rannala and Yang 2017) (fig. 1*a*), whereas in the phylogenetic relaxed-clock models there is no such distinction between the species tree and the gene tree.

We accommodate variation in evolutionary rate both among species and among loci. The among-loci rate variation applies to both strict-clock and relaxed-clock models. Under the relaxed-clock models, we allow among-loci variation both in the overall rate and in the degree of rate variation among species: some loci may have limited among-species rate variation and nearly satisfy a strict clock model, whereas others may have serious rate variation that violates the clock. Important parameters of the MSC model such as species divergence times and population sizes may be estimated jointly. This is the full-likelihood approach, which extracts information available from both gene tree topologies and coalescent times, whereas accommodating their uncertainties due to finite sequence length at each locus and allowing rate variation among species (clock violations).

An alternative approach to accommodating violations of the molecular clock in an MSC framework is to infer unrooted gene trees using phylogenetic methods without assuming a clock and then to use the inferred gene trees as data to estimate the species tree (with internal branch lengths in coalescent units), using an outgroup to root the tree. This is the two-step summary approach, used in MP-est (Liu et al. 2010), NJ${}_{\mathrm{st}}$ (Liu and Yu 2011), and astral (Mirarab and Warnow 2015).

The two-step methods are computationally efficient but they ignore information in the branch lengths (coalescent times) in gene trees. They often treat inferred gene trees as observations without properly accommodating phylogenetic reconstruction errors, although some efforts have been made to account for uncertainties in gene tree topologies (Sayyari and Mirarab 2016). The full-likelihood MSC approach is computationally demanding. Furthermore, when the clock is seriously violated, temporal information from the coalescent times may be eroded even when among-species variations of clock rates are accounted for in the model. One may thus expect the full-likelihood approach to have an advantage over two-step methods when the clock holds or is violated only slightly, but the benefit may diminish with increasing violations of the clock. An advantage of the full-likelihood approach over heuristic two-step methods is that it additionally provides estimates of species divergence times (measured in units of the expected number of substitutions per site), which may be converted to estimates of absolute geological times when the tree is calibrated using information from the fossil record (Angelis 2015; dos Reis et al. 2016). Two-step methods using gene tree topologies can identify internal branch lengths in coalescent units on the species tree but these cannot be directly translated into geological time units. Important parameters in the MSC model, such as external branch lengths on the species tree (or species divergence times) and population sizes for modern and ancestral species are simply not identifiable by those methods (Xu and Yang 2016; Zhu and Yang 2021).

In this paper, we extend the models implemented in bpp to allow deep phylogenetic trees to be analyzed. We incorporate two major changes to the program. First, we implement the GTR+Γ substitution model (Yang 1994a, 1994b) and its special cases, in addition to JC. Second, we relax the strict clock assumption by adapting the relaxed-clock models developed in Bayesian phylogenetics for divergence time estimation (Rannala and Yang 2007) to the MSC framework. We validate our implementation of the methods in bpp and explore the impacts of clock assumptions on estimates of the species tree and MSC model parameters using simulations. We analyze two empirical datasets, one of gibbons (Carbone et al. 2014; Shi and Yang 2018) and another of the flightless birds ratites (Cloutier et al. 2019). The gibbon dataset represents a shallow species tree, with an approximately constant rate of evolution, so we expect that relaxed clocks with GTR+Γ should produce similar results to the early analyses under the clock+JC model (Shi and Yang 2018). The ratite tree represents a deep phylogeny with far more distantly related species and with the molecular clock assumption seriously violated. In such a case, we expect the use of a strict clock could lead to seriously biased estimates, whereas the relaxed clock may be a major improvement.

## Theory

### Overview of MSC+relaxed Clock Model

**Fig. 2.**
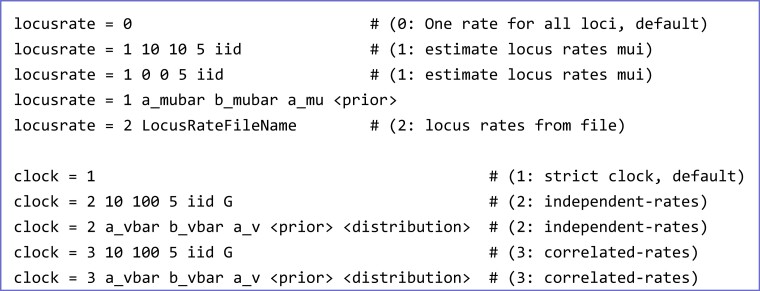
The relaxed-clock models are specified using two control variables in bpp: (i) locusrate concerning the overall rate $\mu_{i}$ for locus i and (ii) clock concerning the rate variance parameter $\nu_{i}$ for locus i. The locusrate variable is used with any of the three clock models (clocks 1, 2, 3). In the example $\alpha_{\bar{\mu }}=10$ and $\beta_{\bar{\mu }}=10$ specify the mean overall rate $\bar{\mu }∼G(\alpha_{\bar{\mu }},\beta_{\bar{\mu }})$. When there are no fossil calibrations on the species tree, $\bar{\mu }=1$ is fixed, specified using $\alpha_{\bar{\mu }}=\beta_{\bar{\mu }}=0$. Given the mean overall rate $\bar{\mu }$, the overall rates for loci ($\mu_{i}$) are generated from the conditional-independence model (iid) or the gamma-Dirichlet model (dir), with the shape parameter $\alpha_{\mu }$ (= 5 in the example) specifying how similar $\mu_{i}$ are among loci. The clock variable specifies the three clock models: clock 1 (strict clock), clock 2 (independent-rates model), and clock 3 (correlated-rates model). Under both clock 2 and clock 3, the average rate variance parameter is specified as $\bar{\nu }∼G(\alpha_{\bar{\nu }},\beta_{\bar{\nu }})$; in the example $\alpha_{\bar{\nu }}=10$ and $\beta_{\bar{\nu }}=100$ with mean 0.1. Given $\bar{\nu }$, the variance $\nu_{i}$ for locus i is similarly generated from the iid or dir models. Given the overall rate $\mu_{i}$ and the rate variance parameter $\nu_{i}$ for locus i, rates for branches at locus i are specified for clock 2 and clock 3 using either the gamma (G) or log-normal (LN) distributions.

We develop MCMC algorithms for Bayesian inference under the MSC model with relaxed clocks for sampling from the joint posterior distribution of species trees, species divergence times, and other parameters of interest. The parameters of the MSC+relaxed clock model are illustrated in figure 1*a*. The model is specified using two variables (locusrate and clock) in the bpp control file (fig. 2).

Let Ψ={T,τ,θ} represent the species tree for s species, with T to be the species tree topology, τ the species divergence times, and θ the (effective) population sizes for all populations on the species tree. Both τ and θ are measured in the expected number of substitutions per site. Let $X=\{X_{i}\}$ be the multilocus sequence data, with $X_{i}$ to be a matrix of aligned sequences for the sampled individuals at locus i, with i=1,…,L. The sequences may be unphased diploid sequences (Flouri et al. 2018; Huang et al. 2022). Let $G=\{G_{i},t_{i}\}$ be the gene trees at the L loci, where $G_{i}$ is the gene tree topology and $t_{i}$ the set of coalescence times at locus i. The gene tree $\left(G_{i},t_{i}\right)$ specifies the probability distribution of the sequence alignment at locus i but is not observed.

We assume that substitution rate varies both among loci and, for each locus, across species-tree branches (fig. 1). Let $\mu_{i}$ be the overall (mean) rate for locus i, and $\nu_{i}$ be the rate variance parameter for locus i, with $\mu =\{\mu_{i}\}$ and $\nu =\{\nu_{i}\}$. Parameter $\nu_{i}$ specifies how fast the rate changes or evolves over time, with a larger $\nu_{i}$ representing faster evolution of the rate or more serious violation of the clock. Given $\mu_{i}$ and $\nu_{i}$ for locus i, the rate evolves among species-tree branches, thus relaxing the clock assumption. Furthermore, we assume that the rates are changing independently among loci (fig. 1). Let $r_{ij}$ be the rate at locus i for species-tree branch j, with $R=\{r_{ij}\}$.

**Fig. 3.**
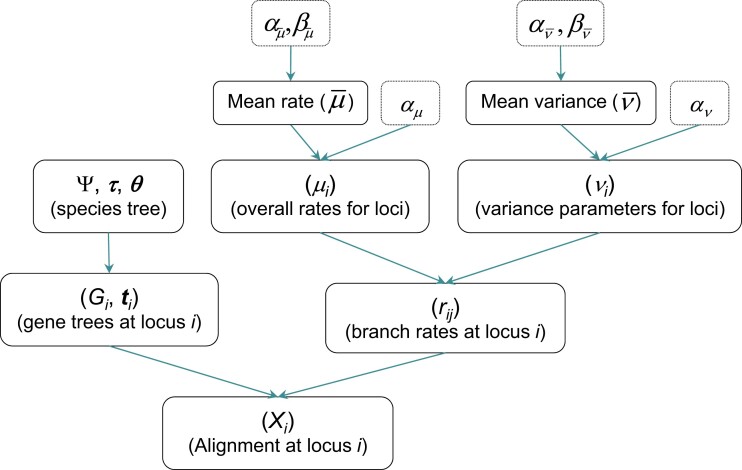
DAG (for directed acyclic graphical model) representation of the MSC+relaxed clock model implemented in this paper, illustrating the conditional independence of different components in the model. The species tree (Ψ) and the parameters on the species tree including species divergence times (τs) and population sizes (θs) specify the probability density of the gene trees at the multiple loci (gene tree topology $G_{i}$ and coalescent times $t_{i}$ for locus i) (Rannala and Yang 2003). The relaxed-clock or rate-evolution model is specified by two components, the overall rates for loci ($\mu_{i}$) and the rate variance parameters for loci ($\nu_{i}$). The overall rates for loci ($\mu_{i}$) are specified using either the gamma-Dirichlet or conditional i.i.d. priors conditioned on the mean overall rate ($\bar{\mu }$). Similarly the variance parameters for loci ($\nu_{i}$) are specified using the gamma-Dirichlet or conditional i.i.d. priors conditioned on the mean variance parameter ($\bar{\nu }$). Given the overall rate $\mu_{i}$ and the variance parameter $\nu_{i}$ for locus i, the species-specific branch rates ($r_{ij}$ for branch j at locus i) are specified using either the independent-rates model (clock 2) or the correlated-rates model, based on either a log-normal or gamma kernel. For each locus i, given the gene tree topology ($G_{i}$), the coalescent times ($t_{i}$), and the branch rates ($r_{ij}$), the branch lengths on the gene tree are specified as the sum of the segments for each branch (fig. 1*a*). Finally, the gene tree topology ($G_{i}$) and branch lengths specify the phylogenetic likelihood (Felsenstein 1981) or allow a sequence alignment for the locus to be simulated.

We assign a prior on the locus rates μ with parameters $\Omega_{\mu }=\{\alpha_{\mu },\alpha_{\bar{\mu }},\beta_{\bar{\mu }}\}$, and a prior on the rate variance parameters ν with parameters $\Omega_{\nu }=\{\alpha_{\nu },\alpha_{\bar{\nu }},\beta_{\bar{\nu }}\}$. Let Θ include parameters in the prior for MSC model parameters (τ and θ). $\Omega_{\mu },\Omega_{\nu }$ and Θ are parameters in the priors or hyper-priors, specified by the user. The MCMC samples from the joint posterior density

(1) $$\begin{array}{c} f(\Psi ,\mu ,v,R,G|X,\Omega_{\mu },\Omega_{v},\Theta )\\ \alpha f(X|G,R)f(G|\Psi )f(R|\Psi ,\mu ,v)\\ \times f(\Psi |\Theta f(\mu |\bar{\mu },\alpha_{\mu })f(\bar{\mu }|\alpha_{\bar{\mu }},\beta_{\bar{\mu }})\\ \times f(v|\bar{v},\alpha_{v})f(\bar{v}|\alpha_{\bar{v}},\beta_{\bar{v}}), \end{array}$$

where f(X|G,R) is the probability of the sequence alignments given the gene trees and branch lengths or the so-called phylogenetic likelihood (Felsenstein 1981), f(G|Ψ) is the density of the gene tree under the MSC (Rannala and Yang 2003), f(R|Ψ,μ,ν) is the probability density of branch rates, and the remaining terms are priors (and hyper-priors) in the rate-evolution model. The conditional independence of components in the model is illustrated in figure 3.

### Overall Rate Parameter for a Locus

We implemented two choices for the prior probability distribution of the overall substitution rates, $\mu =\{\mu_{i}\}$, at the L loci: the *gamma-Dirichlet* (dir) prior (Burgess and Yang 2008; dos Reis et al. 2014) and the *conditional i.i.d.* (iid, for identically and independently distributed) prior (Zhu and Yang 2015) (table 1). Both prior models make use of the mean overall rate across all loci, $\bar{\mu }$, which is treated in two ways. If there are fossil calibrations on the species tree, to allow estimation of absolute species divergence times and absolute substitution rates, we assign a gamma hyper-prior, $\bar{\mu }∼G(\alpha_{\bar{\mu }},\beta_{\bar{\mu }})$, with density

(2) $$f(\bar{\mu }\;|\;\alpha_{\bar{\mu }},\beta_{\bar{\mu }})=\frac{\beta_{\bar{\mu }}^{\alpha_{\bar{\mu }}}}{\Gamma (\alpha_{\bar{\mu }})}{\bar{\mu }}^{\alpha_{\bar{\mu }}-1}\;e^{-\beta_{\bar{\mu }}\bar{\mu }}.$$

Alternatively, if no fossil calibrations are available, in which case the rates are relative and species divergence times are measured in the expected number of substitutions, we fix $\bar{\mu }=1$ (specified by $\alpha_{\bar{\mu }}=\beta_{\bar{\mu }}=0$; fig. 2). Analysis in this paper use this second formulation.

**Table 1.** Parameters in the Prior Model for the Overall Locus Rates ($\mu_{i}$) and Rate Variance Parameters ($\nu_{i}$) for L Loci.

Model	No. parameters	Parameters
Gamma-Dirichlet (dir)	2L	$\mu_{1},\mu_{2},\ldots ,\mu_{L}$
		$\nu_{1},\nu_{2},\ldots ,\nu_{L}$
Conditional i.i.d. (iid)	2(L+1)	$\mu_{1},\mu_{2},\ldots ,\mu_{L},\bar{\mu }$
		$\nu_{1},\nu_{2},\ldots ,\nu_{L},\bar{\nu }$

Note.—Under the Gamma-Dirichlet model, $\bar{\mu }=(1/L)\sum_{i=1}^{L}\mu_{i}$ and $\bar{\nu }=(1/L)\sum_{i=1}^{L}\nu_{i}$ are printed out by bpp, but they are not free parameters in the model.

In the *gamma-Dirichlet* (dir) prior, the total rate $L\bar{\mu }=\sum_{i}\mu_{i}$ is partitioned into rates for loci ($\mu_{i}$) according to a Dirichlet distribution with concentration parameter $\alpha_{\mu }$. Smaller values of $\alpha_{\mu }$ mean greater variation in rates among loci. The joint density of the L locus rates, $\mu =(\mu_{i})$, is

(3) $$\begin{array}{rl} & f(\mu \;|\;\alpha_{\bar{\mu }},\beta_{\bar{\mu }},\alpha_{\mu })=\frac{(\beta_{\bar{\mu }}/L{)}^{\alpha_{\bar{\mu }}}}{\Gamma (\alpha_{\bar{\mu }})}\cdot \frac{\Gamma (L\alpha_{\mu })}{\Gamma (\alpha_{\mu }{)}^{L}}\\ & \;\times {\left(\sum_{i=1}^{L}\mu_{i}\right)}^{\alpha_{\bar{\mu }}-L\alpha_{\mu }}\times e^{-\beta_{\bar{\mu }}\sum \mu_{i}/L}\;{\left(\prod_{i=1}^{L}\mu_{i}\right)}^{\alpha_{\mu }-1} \end{array}$$

(dos Reis et al. 2014, eq. 5; see also Burgess and Yang 2008). In the *conditional i.i.d.* (iid) prior the overall rate $\mu_{i}$ for locus i has a gamma distribution G$\left(\alpha_{\mu },\alpha_{\mu }/\bar{\mu }\right)$ with shape parameter $\alpha_{\mu }$ and mean $\bar{\mu }$, so that the joint prior for μ is

(4) $$f(\mu \;|\;\bar{\mu },\alpha_{\mu })=\prod_{i=1}^{L}f(\mu_{i}\;|\;\alpha_{\mu },\alpha_{\mu }/\bar{\mu }).$$

In this model, the rates $\mu_{i}$ at the L loci are parameters, so the distribution is L-dimensional (table 1).

Note that in both the gamma-Dirichlet and conditional i.i.d. models, $\alpha_{\mu }$ and $\alpha_{\bar{\mu }}$ are distinct parameters: $\alpha_{\bar{\mu }}$ specifies how certain we are about the average rate ($\bar{\mu }$), with a larger $\alpha_{\bar{\mu }}$ meaning more confidence, whereas $\alpha_{\mu }$ specifies how similar the overall rates ($\mu_{i}$) are among loci, with a larger $\alpha_{\mu }$ meaning highly similar rates among loci.

### Rate Variance Parameter for a Locus

We also implemented two prior distributions for the variance parameter $\nu_{i}$ for locus i: the *gamma-Dirichlet* (dir) prior and the *conditional i.i.d.* (iid) prior. For both priors, the average variance parameter across all loci $\bar{\nu }$ is assigned a gamma hyper-prior, $\bar{\nu }∼G(\alpha_{\bar{\nu }},\beta_{\bar{\nu }})$, with density

(5) $$f(\bar{\nu }\;|\;\alpha_{\bar{\nu }},\beta_{\bar{\nu }})=\frac{\beta_{\bar{\nu }}^{\alpha_{\bar{\nu }}}}{\Gamma (\alpha_{\bar{\nu }})}{\bar{\nu }}^{\alpha_{\bar{\nu }}-1}\;e^{-\beta_{\bar{\nu }}\bar{\nu }}.$$

In the *gamma-Dirichlet* prior the sum $L\bar{\nu }=\sum_{i}\nu_{i}$ is partitioned into $\nu_{i}$ for loci according to a Dirichlet distribution with concentration parameter $\alpha_{\nu }$. Smaller values of $\alpha_{\nu }$ mean greater variation in $\nu_{i}$ among loci (e.g., the clock is seriously violated at some loci but not at others). The joint density of the L locus-specific rate-evolution parameters is thus

(6) $$\begin{array}{rl} & f(\nu \;|\;\alpha_{\bar{\nu }},\beta_{\bar{\nu }},\alpha_{\nu })=\frac{(\beta_{\bar{\nu }}/L{)}^{\alpha_{\bar{\nu }}}}{\Gamma (\alpha_{\bar{\nu }})}\cdot \frac{\Gamma (L\alpha_{\nu })}{\Gamma (\alpha_{\nu }{)}^{L}}\\ & \;\times {\left(\sum_{i=1}^{L}\nu_{i}\right)}^{\alpha_{\bar{\nu }}-L\alpha_{\nu }}\times e^{-\beta_{\bar{\nu }}\sum \nu_{i}/L}\;{\left(\prod_{i=1}^{L}\nu_{i}\right)}^{\alpha_{\nu }-1}. \end{array}$$

In the *conditional i.i.d.* model the rate variance parameter for locus i is assigned a gamma prior, $\nu_{i}\;|\;\bar{\nu }∼G(\alpha_{\nu },\alpha_{\nu }/\bar{\nu })$ so the joint density is

(7) $$f(\nu \;|\;\bar{\nu },\alpha_{\nu })=\prod_{i=1}^{L}f(\nu_{i}\;|\;\alpha_{\nu },\alpha_{\nu }/\bar{\nu }).$$

In both priors for $\nu_{i}$, $\alpha_{\bar{\nu }}$ specifies our certainty about the average rate variation among lineages ($\bar{\nu }$). For closely related species, we expect the molecular clock to hold approximately for every locus, so we could specify a large $\alpha_{\bar{\nu }}$ and a small mean $\alpha_{\bar{\nu }}/\beta_{\bar{\nu }}$ (e.g., $\alpha_{\bar{\nu }}=10$ and $\beta_{\bar{\nu }}=1000$ with mean 0.01). Conversely, the concentration parameter $\alpha_{\nu }$ specifies the degree of similarity among loci in terms of their clock violation. Larger $\alpha_{\nu }$ (e.g., 10 or 100) may be used if clock violations are similar among loci, whereas small values (e.g., 1) may be used if the clock is seriously violated at some loci but not others.

### Rates for Branches at a Locus

Our MSC+relaxed clock model assigns rates at any locus to branches on the species tree, rather than on gene trees (fig. 1*a*) (Xu and Yang 2016). A gene-tree branch may pass through multiple species and comprises multiple segments with different rates, and the branch length is calculated by summing over the segments, with each segment length being a product of the rate and the time duration. In contrast, multiple branches on a gene tree may all reside in a single species and have the same rate. For example, if all sequences at a locus are sampled from the same species and all coalescent events occur in that species (before reaching an ancestral species), all branches on the gene tree will have the same rate even if the relaxed-clock model allows different rates among species.

Given the overall rate $\mu_{i}$ and the rate variance parameter $\nu_{i}$ at locus i, the branch rate $r_{ij}$ (for species-tree branch j at locus i) is defined as the rate for the mid-branch and applies to the whole time duration of the population. For example, the rate for branch A in figure 1*a* is the rate for the mid-point of branch A and applies to population A over its whole time duration $\left(0,\tau_{AB}\right)$. We implement two models to describe the rate-evolution process: the *independent-rates* (clock 2) and the *correlated-rates* (clock 3) models. For each, we used either a gamma or log-normal kernel. Note that the root branch (stem) of the species tree has a rate as well, which applies to gene-tree branches residing in that species.

The *independent-rates model* assumes 2s−1 independent branch rates at every locus. Although a rooted species tree for s species has 2s−2 branches, we have 2s−1 branch rates, including a rate for the root branch on the species tree. The joint density for the branch rates is

(8) $$f(R\;|\;\mu ,\nu ,\Psi )=\prod_{i=1}^{L}\prod_{\;j=1}^{2s-1}f(r_{ij}\;|\;\mu_{i},\nu_{i}),$$

where the density f is either the gamma or log-normal. Under the independent gamma model,

(9) $$r_{ij}\;|\;\mu_{i},\nu_{i}∼G\left(\frac{\mu_{i}^{2}}{\nu_{i}},\frac{\mu_{i}}{\nu_{i}}\right),$$

which has mean $\mu_{i}$ and variance $\nu_{i}$. Under the independent log-normal model,

(10) $$r_{ij}\;|\;\mu_{i},\nu_{i}∼\mathrm{LN}(\mu_{i},\nu_{i}),$$

with density

(11) $$f(r_{ij}\;|\;\mu_{i},\nu_{i})=\frac{1}{r_{ij}\sqrt{2\pi \nu_{i}}}\exp\;\left\{-\frac{1}{2\nu_{i}}{\left(\log\;\frac{r_{ij}}{\mu_{i}}+\frac{1}{2}\nu_{i}\right)}^{2}\right\},\;0<r_{ij}<\infty .$$

This has mean $\mu_{i}$ and variance $(e^{\nu_{i}}-1)\mu_{i}^{2}$. Note that $\mu_{i}$ is the mean of the rate (rather than the mean of the log rate), whereas $\nu_{i}$ is the variance of the log rate (rather than the variance of the rate). The bias-correction term, $\frac{1}{2}\nu_{i}$, was introduced by Kishino et al. (2001) to ensure that the distribution has the mean $\mu_{i}$.

The *correlated-rates* model specifies rates for daughter branches conditional on the rate for the mother branch, thus introducing correlation between branches. Rates are assigned to the midpoints of branches on the species tree and apply to the time duration of the population represented by the branch. The overall rate $\mu_{i}$ for locus i is also used as the rate for the root population at the locus. With this formulation, the correlated-rates model has L fewer parameters than the independent-rates model (which uses rates for the root branch at the L loci, distinct from $\mu_{i}$). Again we implement both the gamma (G) and log-normal (LN) distributions of rates for the daughter branches given the parental rate (fig. 2).

The *correlated log-normal* model specifies the geometric Brownian motion model of Thorne et al. (1998) and Kishino et al. (2001), modified by Rannala and Yang (2007) to account for the correlation in rates between the two daughter branches due to shared rate evolution. There are 2s−2 branch rates at each locus, and their joint density is

(12) $$\begin{array}{rl} & f(R\;|\;\mu ,\nu ,\tau )=\prod_{i=1}^{L}\prod_{\;j=1}^{s-1}f(r_{ic_{\;j1}},r_{ic_{\;j2}}\;|\;r_{ia_{j}},\mu_{i},\nu_{i},\tau ), \end{array}$$

where $a_{j}$ is the jth mother branch, and $c_{\;j1}$ and $c_{\;j2}$ are its two child branches. For each locus, the product is over the s−1 internal nodes on the species tree, with the distributions of the branch rates specified recursively starting at the root. Given the rate at the species-tree root $\mu_{i}$, the rates for its two daughter branches are specified. Then given the rate for each parental branch, the rates for its two daughter branches are specified by integrating over the rate at the internal node that is ancestral to the daughter branches (Rannala and Yang 2007, eq. 7). For example, given the rate $r_{AB}$ for the parental branch AB in figure 1*a*, the rates for the two daughter branches $r_{A}$ and $r_{B}$ have a bivariate log-normal density $f(r_{A},r_{B}\;|\;r_{AB};\nu_{i})$, where $\nu_{i}$ is the rate variance parameter at locus i. This has mean $E(\begin{array}{c} r_{A}\\ r_{B} \end{array})=(\begin{array}{c} r_{AB}\\ r_{AB} \end{array})$ and correlation depending on both $\nu_{i}$ and the lengths of the daughter branches ($\tau_{AB}$). In other words, given $r_{AB}$, the rates $r_{A}$ and $r_{B}$ are correlated because both evolved from the same rate at the ancestral node AB, and the correlation is ≈1 when $\tau_{AB}\approx 0$ and becomes weaker when $\tau_{AB}$ increases. The probability density of the rates for the whole tree is calculated using a pre-order tree traversal, starting from the root moving towards the tips, until all branches are visited.

The *correlated gamma model* has the joint density of the rates for the 2s−1 branches as

(13) $$f(R\;|\;\mu ,\nu ,\tau )=\prod_{i=1}^{L}\prod_{\;j=2}^{2s-1}f(r_{ij}\;|\;r_{ia},\mu_{i}),$$

where $r_{ia}$ is the rate for the branch ancestral to j at locus i. We specify the rates for species-tree branches recursively, starting from the root and moving towards the tips. The species-tree root has rate $\mu_{i}$ at locus i. Then given the rate for each parental branch $r_{ia}$, the rates for its two daughter branches $r_{i1}$ and $r_{i2}$ are independent gamma variables with mean $r_{ia}$ and variance $\nu_{i}$:

(14) $$r_{ij}\;|\;r_{ia},\nu_{i}∼G\left(\frac{r_{ia}^{2}}{\nu_{i}},\frac{r_{ia}}{\nu_{i}}\right),\;j\in \{1,2\}.$$

Our correlated-gamma model assumes conditional independence of the daughter rates given the parental rate (eq. 14) and fails to account for the correlation between daughter rates due to shared evolution (e.g., both daughter rates $r_{A}$ and $r_{B}$ in fig. 1*a* evolved from the same rate at the node AB). This is an empirical model with no mechanistic basis, unlike the correlated log-normal model, which describes the geometric Brownian motion (GBM) process.

Note that the rate variance parameter $\bar{\nu }$ has different interpretations in clock 2 and clock 3 and between the gamma and log-normal distributions. Brown and Yang (2010) noted that $\bar{\nu }=0.1$ in the correlated log-normal model means fairly strong violation of the clock; the clock is easily rejected by a likelihood ratio test in data simulated at $\bar{\nu }=0.1$.

### Priors for τ and θ

As in previous versions of bpp a prior was placed on the root age on the species tree with the remaining node ages (τs) following a Dirichlet distribution conditional on the root age. Two choices of prior were implemented for the root age ($\tau_{0}$): a gamma prior G(α,β) with mean α/β and an inverse-gamma prior invG(α,β) with mean β/(α−1). Three choices of prior were implemented for θ: an inverse-gamma prior invG(α,β) with mean β/(α−1) (first introduced in bpp3, Yang 2015); a gamma prior G(α,β) with mean α/β, and a beta prior beta(α,β,a,b), with shape parameters α and β, in the range a<θ<b, and with mean (αb+βa)/(α+β). The inverse-gamma prior allows the θ parameters to be integrated out analytically but has the disadvantage that it is heavy-tailed, which can cause mixing problems. The beta density allows a hard upper bound to be placed on θ which could also improve mixing.

### Outgroups and Constraints on Species Tree Topology

With deep phylogenetic trees and among-species rate variation explicit inclusion of outgroup species in the data may add phylogenetic information, although the information may decrease with increased rate variation among species. We therefore implemented topological constraints on species trees during species tree search (A01, Yang 2015). Constraints are specified by defining clades using the constraint and outgroup keywords, the latter of which means that the ingroup species form a clade. Note that species trees are always rooted in bpp, under both the strict-clock and relaxed-clock models.

### Extension of the Nucleotide Substitution Model

The mutation/substitution model in bpp is extended from JC (Jukes and Cantor 1969) to GTR+Γ (Yang 1994a, 1994b). Standard priors are assigned to the parameters of the model and proposals are implemented to modify them in the MCMC algorithm (Yang 2014, Chapter 8). A uniform Dirichlet prior is assigned to the base frequencies ($\pi_{T},\pi_{C},\pi_{A},\pi_{G}$) and another uniform Dirichlet prior is assigned to the ‘exchangeability’ parameters (a,b,c,d,e,f) of the GTR model (Yang 1994a). A gamma prior is assigned to the shape parameter α for gamma-distributed rates among sites (Yang 1994b). Simpler models that are special cases of GTR+Γ are implemented as well, including K80 (Kimura 1980) and HKY (Hasegawa et al. 1984, 1985).

### Implementation of the MCMC Algorithms

We modified the subtree-pruning-and-regrafting (SPR) algorithm for proposing changes to the species tree (Yang and Rannala 2014; Rannala and Yang 2017) under the MSC+relaxed clock models. An example is illustrated in figure 1 for the case of three species, in which case the SPR move is equivalent to the nearest-neighbor-interchange (NNI) move. The move keeps the MSC parameters (τs and θs) unchanged, and prunes off and regrafts so-called affected nodes on the gene trees to avoid conflicts with the proposed species tree, keeping the coalescent times unchanged during the move (fig. 1) (Yang and Rannala 2014). Thus, the MSC density of gene tree and coalescent times may not change in the move, but the likelihood for the sequence alignments may change. As an extreme example, for the species trees S and $S^{\prime }$ of figure 1, suppose the gene tree is [(a,b),c] with both inner nodes to reside in the root species ABC so that there are no affected nodes. Although the gene tree topology and coalescent times remain unchanged during the move, the branch lengths and the likelihood change, due to the mapping of the branch-rates in the relaxed-clock model.

### Validation of the MCMC Algorithms

We have conducted various tests to validate our implementation of the MCMC algorithm (Yang 2014, pp. 241–242). We ran bpp with the likelihood set to 1 to confirm that the MCMC samples from the prior distribution of the parameters, and the gene tree topologies and coalescent times match the expected distributions. This test was effective during the early stages of debugging. See the supplementary text, Supplementary Material online for details.

We conducted a Bayesian simulation to confirm the expectation that when model parameters are sampled from the prior and used to simulate data, the posterior matches the prior. From

(15) f(X)f(Θ|X)=f(Θ)f(X|Θ),

we have

(16) ∫f(X)f(Θ|X)dX=∫f(Θ)f(X|Θ)dX=f(Θ).

Thus any expectation of the prior distribution can be written as an average over the replicate datasets and, for each dataset, over the posterior distribution:

(17) $$\begin{array}{rl} h & =\int h(\Theta )\;f\;(\Theta )\;d\Theta \\ & =\iint h(\Theta )\;f\;(X)\;f\;(\Theta \;|\;X)\;dX\;d\Theta \\ & \approx \frac{1}{R}\sum_{i=1}^{R}\int h(\Theta )\;f\;(\Theta \;|\;X_{i})\;d\Theta ,\\ & \approx \frac{1}{RN}\sum_{i=1}^{R}\sum_{t=1}^{N}h(\Theta_{t}^{\left(i\right)}), \end{array}$$

where $\Theta_{t}^{\left(i\right)},\;t=1,\ldots ,N$ are an MCMC sample from the posterior $f(\Theta |X_{i})$ in the analysis of replicate dataset $X_{i}$, whereas the function h(Θ) is calculated using the sampled values from the posterior. Each replicate dataset ($X_{i}$) is generated by sampling parameters Θ from the prior f(Θ) and then simulating under the likelihood model f(X|Θ) using those parameter values. Each dataset is then analyzed to generate the posterior $f(\Theta \;|\;X_{i})$ and to calculate the posterior mean of h(Θ). By averaging over R replicate datasets and over N MCMC samples for each dataset, we recover the prior expectation h.

Here the function h(Θ) is generic. If h(Θ)=ϕ, where ϕ is any scalar parameter (such as $\tau_{0}$, the age of the species-tree root), h will be the mean of the distribution. If $h(\phi )=I_{\phi_{l}<\phi <\phi_{u}}$, then $h=P\{\phi_{l}<\phi <\phi_{u}\}$ will be the probability that ϕ falls in the fixed interval $\left(\phi_{l},\phi_{u}\right)$. If $h(\phi )=I_{\phi <a}$, then h=P{ϕ<a} will be the cumulative density function (CDF). Note that the function h(Θ) can be multivariate, allowing the estimation of joint densities, even though here we focus on marginal distributions only.

Equation (17) holds for any fixed size, L, of the dataset X (in the current context, L is the number of loci). If L=0, the posterior distribution for each dataset will match the prior, and equation (17) will not constitute a useful test. If L is very large, however, analysis of each dataset by MCMC will be more expensive and furthermore the posterior distribution for each dataset will be highly concentrated so that more replicate datasets (large R) may be needed to produce a smooth estimation of the average posterior density. Note that when L→∞, the posterior distribution for each dataset degenerates to a point mass at the true parameter value. In sum, ideally the datasets should be small enough to avoid heavy computation but large enough so that the posterior distribution for each parameter is influenced by both the prior and the data likelihood. It is advisable to plot the posterior densities for replicate datasets to confirm that they are different (i.e., X is sufficiently large so that the posterior is influenced by the data). In our analyses, 10 loci were used in each dataset. Some authors have advocated the use of a formal statistical test to evaluate the difference between the average posterior and the prior (e.g., Cook et al. 2006). However, failure to detect a difference with a formal test could be due to either the low power of the test or small sample size (small R and N in eq. 17) and may not indicate a genuine match between the average posterior and the prior or the correctness of the MCMC algorithm. Thus to ensure that any test has nearly 100% power, a very large number of simulated datasets (R) may be necessary, and furthermore, the impact of L needs to be considered.

We let Θ represent both the parameters in the MSC+relaxed clock model and the gene trees (which are latent variables in the model). We let X represent the data of multi-locus sequence alignments, and use equation (17) to recover the whole prior distribution via simulation. The prior distributions for some parameters are analytically available: for example, the age of the root of the species tree ($\tau_{0}$) and the θ parameters are assigned independent gamma priors, and the GTR exchangeability parameters a,b,c,d,e,f are assigned a Dirichlet prior. The prior distributions for other parameters, and for the gene trees, may be intractable analytically but can be estimated numerically by sampling parameters from the prior f(Θ) and simulating the gene trees from f(G|Θ). These methods were used to conduct a Bayesian simulation to validate our bpp implementation of the MSC-relaxed clock models using a species tree for three species. See the supplementary text, Supplementary Material online for details.

We also simulated datasets under relaxed clock models and confirmed that the Bayesian estimates converged to the true values when the data size (the number of loci) increases. See the Results section for more details.

Although we were able to validate the correctness of all three clock models for small datasets, we encountered serious mixing problems in large datasets, in particular for species tree estimation under the correlated-rates model (clock 3). Our analysis of the two real datasets thus relied on the independent-rates model (clock 2). MCMC mixing problems are discussed below in the Results section.

## Results

### Validation of the MCMC Algorithms

We present two sets of test results to validate our implementation in bpp of the MCMC algorithms under MSC-relaxed clocks. In the first set, we ran bpp with the likelihood fixed at 1 to confirm that the posterior distribution of the parameters, which bpp samples from, matches the prior. We used the species tree [(A,B),C] (fig. 1) and monitored 12 parameters in the MSC-relaxed clock model, for which the prior marginals are analytically available for comparison. These include $\theta_{A}$, $\theta_{AB}$, $\theta_{ABC}$, $\tau_{ABC}$, $\tau_{AB}$, and the locus-specific substitution parameters in the GTR+$\Gamma_{5}$: the exchangeability rates a,b,c,d,e,f and the gamma shape parameter α. We used both the independent-rates (clock 2) and correlated-rates (clock 3) models, and for each examined four model settings, with different prior distributions of the overall rate and variance parameters ($\mu_{i}$ and $\nu_{i}$) among loci (conditional i.i.d. versus gamma-Dirichlet), and different kernel distributions of branch rates (gamma versus log-normal). Close matches were observed between the prior and the posterior in each of the eight settings (supplementary figs. S1 and S2, Supplementary Material online).

In the second set of tests, we conducted a Bayesian simulation, generating 200 replicate datasets, each of 10 loci, with each dataset simulated by using parameter values sampled from the prior, and then analyzing the datasets using bpp. Averaged over the replicate datasets, the posterior of parameters is expected to match the prior (see eq. 17). This is a stringent test, and validates both the simulation program and the inference program.

We used the same species tree for three species and the same eight relaxed-clock settings as in the first test. Besides the 12 parameters mentioned above, we monitored four additional locus-specific parameters: $\mu_{1}$ (the overall rate at locus 1), $\nu_{1}$ (the variance parameter at locus 1), and gene-tree tree height (TH or the gene-tree root age) and tree length (TL or the sum of branch lengths). The branch length on the gene tree is calculated as a sum over the different segments and is a function of species divergence times (τs), coalescent times (ts), and branch rates for the locus (fig. 1*a*). In this test, we estimated the priors empirically using sampled values in the simulation even if their analytical forms may be available. See the supplementary SI text, Supplementary Material online for the details of the procedure. Supplementary figures S3 and S4, Supplementary Material online show the prior and average posterior densities for clock 2 and clock 3, respectively, with excellent match as expected from theory. Note that the posterior varies among replicate datasets (supplementary figs. S5 and S6, Supplementary Material online), because the datasets are generated by using different parameter values and because the datasets have a finite size so that the posterior is influenced by the random sampling errors due to the finite data size. However, by averaging over replicate datasets one recovers the prior distribution. The Bayesian simulation tests both the simulation and inference components of the bpp program.

### Simulation to Evaluate Species Tree Estimation

**Fig. 4.**
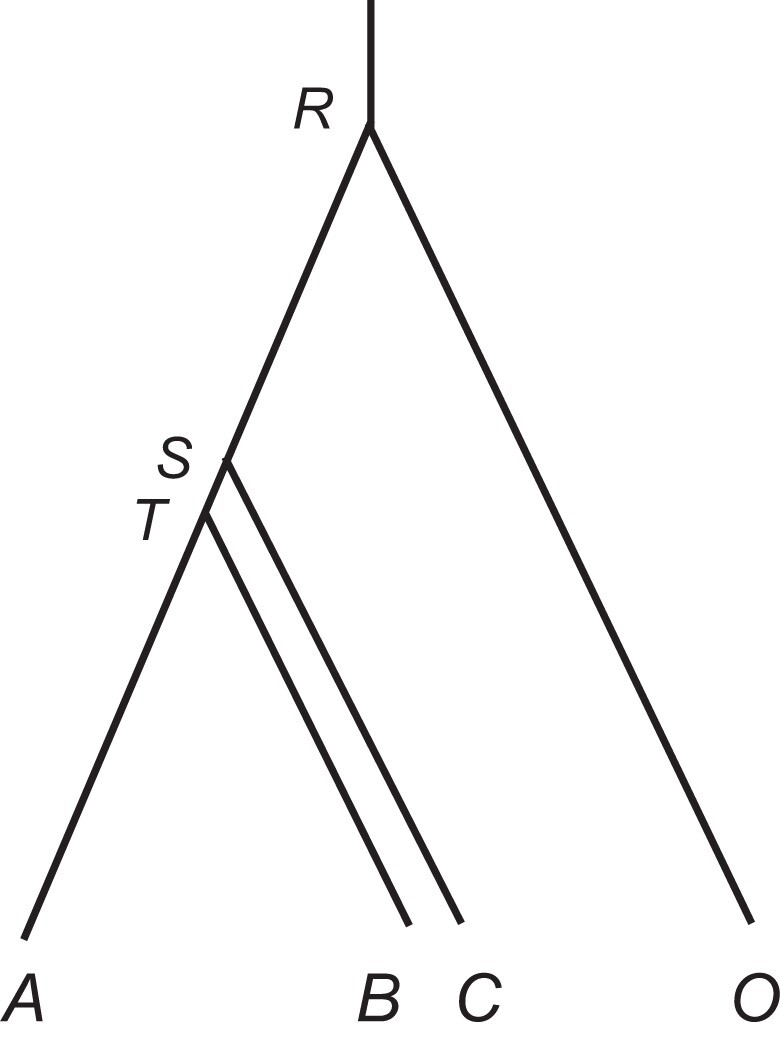
A species tree for four species (A,B,C, with O to be the outgroup) used to simulate multilocus sequence data for species tree estimation. The MSC parameters used are $\tau_{R}=0.2$, $\tau_{S}=0.105,\tau_{T}=0.1$, $\theta_{R}=\theta_{S}=0.01$, and $\theta_{T}=0.05$.

We simulated multilocus sequence data under the MSC+relaxed clock model and analyzed them using bpp, in comparison with astral and MP-est. The species tree of figure 4 was used. Species O was used as the outgroup to root the tree in astral and MP-est, whereas the bpp analysis used either the three ingroup species only or all four species, in which case species O was specified as the outgroup. Note that bpp operates on rooted species trees under both the strict- and relaxed-clock models so that an outgroup is not required. Data were simulated under the GTR+$\Gamma_{5}$ substitution model, whereas both JC and GTR+$\Gamma_{5}$ models were used in the analysis. We used the independent-rates model (clock 2) to simulate data, with two values for the average rate variance parameter: $\bar{\nu }=0.01$ representing slight clock violation, whereas $\bar{\nu }=0.1$ serious clock violation. All three clock models were used for data analyses. This is the A01 analysis (Yang 2015), with the SPR algorithm (Rannala and Yang 2017) used to move between species trees generating a posterior distribution. The maximum *a posteriori* probability (MAP) tree is the Bayesian estimate of the true species tree and its posterior probability is a measure of confidence in the estimate. The results are summarized in table 2.

**Table 2.** Probability (estimated using 100 simulated replicates) that the True Species Tree is Recovered by bpp under Different Clock Models and by astral and MP-est.

			GTR+Γ			JC				
$\bar{\nu }$	LR	Loci	C1	C2	C3	C1	C2	C3	Ast	MP
*With outgroup*										
0.01	No	10	54	55	56	52	51	53	50	44
0.01	No	20	51	50	46	49	50	48	44	43
0.01	No	100	77	77	33	74	74	54	71	70
0.01	No	200	87	87	44	75	75	50	64	65
0.01	Yes	10	45	44	45	51	52	47	45	46
0.01	Yes	20	53	54	48	47	48	42	39	36
0.01	Yes	100	77	81	46	71	71	51	52	52
0.01	Yes	200	81	86	40	79	81	46	66	65
0.1	No	10	35	39	40	39	43	46	44	42
0.1	No	20	39	53	48	46	44	42	46	45
0.1	No	100	50	63	35	48	51	43	52	52
0.1	No	200	72	84	51	66	75	48	75	76
0.1	Yes	10	47	46	47	43	47	45	40	38
0.1	Yes	20	49	54	56	49	52	50	52	51
0.1	Yes	100	59	72	40	63	70	43	54	55
0.1	Yes	200	69	76	44	66	72	46	61	63
*Without outgroup*										
0.01	No	10	47	49	43	47	47	44		
0.01	No	20	48	47	48	50	49	43		
0.01	No	100	58	56	33	65	60	35		
0.01	No	200	78	78	42	78	78	52		
0.01	Yes	10	45	41	46	48	45	49		
0.01	Yes	20	52	53	56	53	53	52		
0.01	Yes	100	69	71	41	69	69	47		
0.01	Yes	200	75	73	47	77	75	34		
0.1	No	10	33	36	35	30	36	36		
0.1	No	20	39	40	40	39	39	41		
0.1	No	100	41	43	33	40	39	27		
0.1	No	200	58	65	43	58	65	43		
0.1	Yes	10	45	44	43	43	44	43		
0.1	Yes	20	38	42	36	38	40	36		
0.1	Yes	100	47	51	37	49	54	27		
0.1	Yes	200	52	56	37	51	57	35		

Note.—Data were simulated under the independent-rates model (clock 2) with and without locus-rate variation (LR) using the four-species tree of figure 4. One sequence was sampled per species per locus. The simulation options are clock = 2 $\bar{\nu }$ 5 iid g (where $\bar{\nu }=0.01$ or 0.1), and locusrate = 1 5 iid. The data were analyzed using bpp to infer the species tree under the strict clock (C1 = clock 1), the independent-rates (C2 = clock 2) and the correlated-rates (C3 = clock 3) models, and using astral (Ast) and MP-est (MP). The control files for simulating and analyzing the data using bpp are shown in supplementary figure S10, Supplementary Material online. Results for clock 3 with L≥20 loci are unreliable due to MCMC mixing problems.

As mentioned above, we observed mixing problems for clock 3 (correlated rates) when the data comprised 20 or more loci. Although the results at L=10 loci are similar to those for clock 2, performance under clock 3 was poorer in larger datasets (with L≥20), and sometimes performance even deteriorated when more loci were included in the data (table 2). We suggest that the poor performance in recovering the true species tree under clock 3 is due to mixing difficulties of the MCMC algorithm, and does not reflect the true performance of the inference method. A typical symptom was that different runs of the same analysis produced inconsistent results. We thus disregard the results for clock 3 with L>20.

At $\bar{\nu }=0.01$, the molecular clock holds approximately, and all three clock models are expected to perform well, with perhaps an advantage for clock 1 (strict clock), due to its smaller size (with fewer parameters). This expectation held for clock 1 and clock 2 (independent rates) (table 2). Also clock 1 and clock 2 recovered the true species tree with higher probabilities than the two-step methods astral and MP-est. For example, in the simulation with locus-rate variation at L=200, clock 1 and clock 2 recovered the true species tree in 81% and 86% of the replicates, whereas the proportions were 66% and 65% for astral and MP-est. This may be explained by the fact that bpp uses information in the gene-tree branch lengths (although accommodating their uncertainties), whereas astral and MP-est do not. Note that astral and MP-est should, in theory, be equivalent to one another in the case of three species plus the outgroup with one sequence sampled per species. The observed differences between the two methods (table 2) are due to the different ways in which they treat ties in the estimated gene trees.

When the species are closely related so that the molecular clock holds approximately and the sequences are highly similar, we expect the mutation model to be unimportant and the clock1+JC model to be adequate for inference using bpp, as the role of the mutation model is to correct for multiple hits in the likelihood calculation in bpp (Xu and Yang 2016; Shi and Yang 2018). We examined this expectation by comparing the posterior probabilities for MAP trees inferred under JC and GTR+Γ for datasets simulated under $\bar{\nu }=0.01$ in supplementary figure S7, Supplementary Material online. With outgroup, GTR+Γ recovered the true tree more often than JC, and JC tends to produce posterior probabilities that are too high. Without outgroup, the two models are much more similar. This may be because the sequence divergence levels are far higher when the outgroup is included in the data. The largest average sequence distance between species is approximately $2\tau_{R}+\theta_{R}=0.41$ mutations per site in datasets with outgroup, and ∼0.22 without outgroup (fig. 4). At such high levels of sequence divergence, correction for multiple hits may be important. For comparison, the sequence divergence between any gibbon species and the human outgroup is ∼3.1% and ∼3.6% for coding and noncoding loci, respectively (Shi and Yang 2018, table 7). The results suggest that JC+clock should be adequate for analysis of genomic data from closely related species, in which the molecular clock holds approximately and the between-species sequence divergence is low, within 10–15%, say. Note that here the data were simulated under GTR+Γ, and the JC model was grossly wrong in its goodness of fit to the data.

At $\bar{\nu }=0.1$, the molecular clock assumption is seriously violated, and the strict-clock model (clock 1) is expected to perform more poorly than clock 2 or clock 3. Clock 1 was indeed poorer than clock 2, especially at L=100 or 200 loci (table 2). Also clock 2 recovered the true species tree with higher probabilities than the two-step methods astral and MP-est. For example, in the simulation with locus-rate variation at L=200, clock 2 recovered the true species tree in 76% of replicates, whereas the proportions were 61% and 63% for astral and MP-est, respectively.

Finally the impact of the outgroup is noteworthy. When the outgroup was excluded, bpp performed consistently worse. For example, in the simulation with $\bar{\nu }=0.1$ and with locus-rate variation, clock 2 recovered the true species tree with probability 76% at L=200 when the outgroup was used, but this dropped to 56% when the outgroup was excluded. Even though bpp operates on rooted trees, including an outgroup adds useful phylogenetic information. In particular, outgroups may be expected to provide important information about the placement of the root for the ingroup, and closely related outgroups may be expected to be more informative than distant outgroups. Nevertheless, bpp/clock 2 recovered the true species tree without the outgroup with increasingly higher probability when the number of loci increased from 10 to 200 (table 2), suggesting that the method is statistically consistent. The results confirm that there is valuable phylogenetic information in the gene-tree branch lengths even when the clock is seriously violated, and that the MSC+relaxed clock model can extract that information. Note that the two-step methods (astral and MP-est) cannot produce an estimate of the species tree at all in the case of three species without an outgroup as there is only one unrooted gene tree for three species.

### Simulation to Evaluate Parameter Estimation

**Fig. 5.**
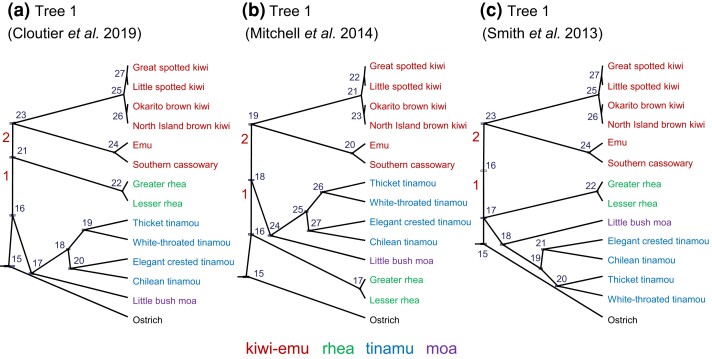
Three species trees for the ratites that differ concerning the placement of the rheas (node 1). Node 2 is in all three trees but received weak support in some analyses (see text). Nodes are numbered to identify parameters in figures 6 & 12. Branches are drawn to represent the posterior means of species divergence times (τ) obtained from bpp analyses of the 250-loci UCE dataset under the independent-rates model (clock 2) accounting for among-loci rate variation (locusrate = 1 0 0 5 dir; clock = 2 2 20 5 dir G).

In the second set of simulations, we examined the performance of bpp for parameter estimation under the MSC+relaxed clock model. We used estimates of MSC parameters obtained from the bpp analysis of the 250 UCE loci for the ratites to simulate biologically realistic datasets under the independent-rates model (clock 2) using species tree 1 of figure 5 both to simulate and to analyze the data (see Materials and Methods). The GTR+$\Gamma_{5}$ model was used to simulate data, with model parameters sampled for each locus.

**Fig. 6.**
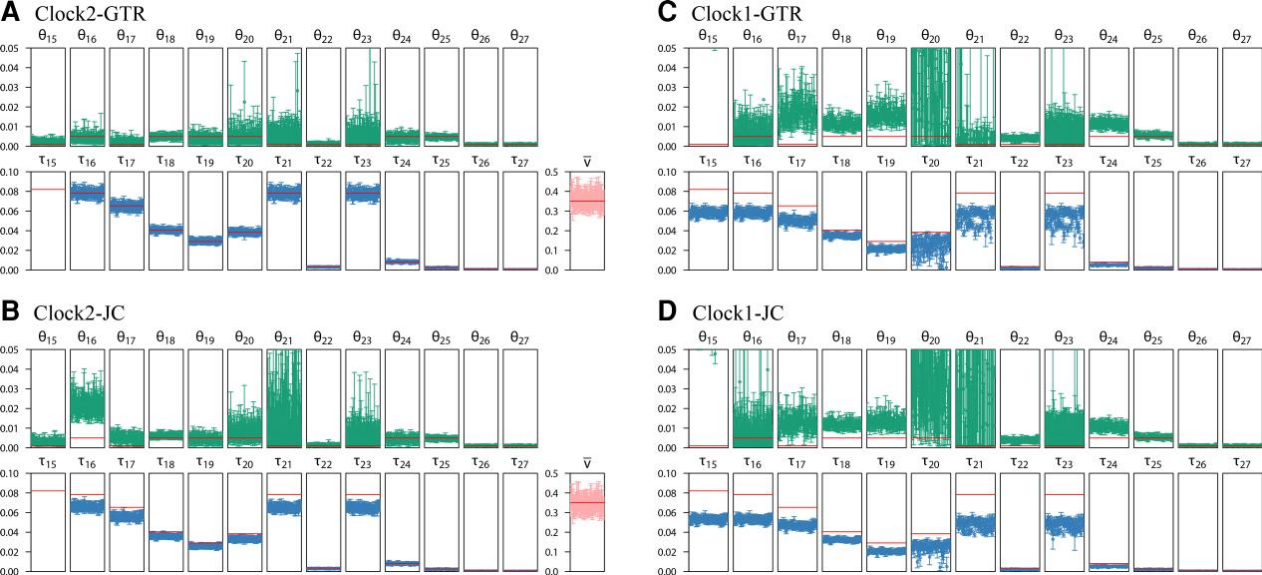
Posterior means and 95% HPD intervals for parameters when the data were simulated under the independent-rates model (clock 2) using parameter estimates from the ratite dataset (tree 1 in fig. 5) and analyzed under either the strict clock (clock 1) or the relaxed clock (clock 2). The species tree was fixed in the bpp analysis. Horizontal lines represent the true values. Note that both τs and θs are measured in the expected number of mutations per site, whereas $\bar{\nu }$ is the rate variance parameter in the rate-evolution model.

The results are summarized in figure 6. Parameters were well estimated under the true model: clock2+GTR+Γ, although the population size parameters for ancestral species corresponding to short branches on the species tree ($\theta_{20},\theta_{21},\theta_{23}$, etc.) had large 95% highest probability density (HPD) credibility intervals (CIs). In particular, all species divergence times were well estimated, with the HPD CIs including the true values. Note that here the replicate datasets are simulated using fixed parameter values, so we are evaluating the Frequentist properties of a Bayesian estimation method. The results are similar to those from the simulation under the strict clock of Huang et al. (2020), in which it was found that the coverage probability of the Bayesian CI exceeds the nominal 95% for well estimated parameters.

Assuming either the strict clock or the JC substitution model led to biased parameter estimates; in particular, species divergence times were seriously underestimated. The incorrect assumption of the strict clock had greater impact than the incorrect assumption of JC, with the biases being the greatest in the JC+clock setting (fig. 6*a*).

### Analysis of the Gibbon Datasets

#### Species Tree Estimation under Different Clock Models and Priors

**Fig. 7.**
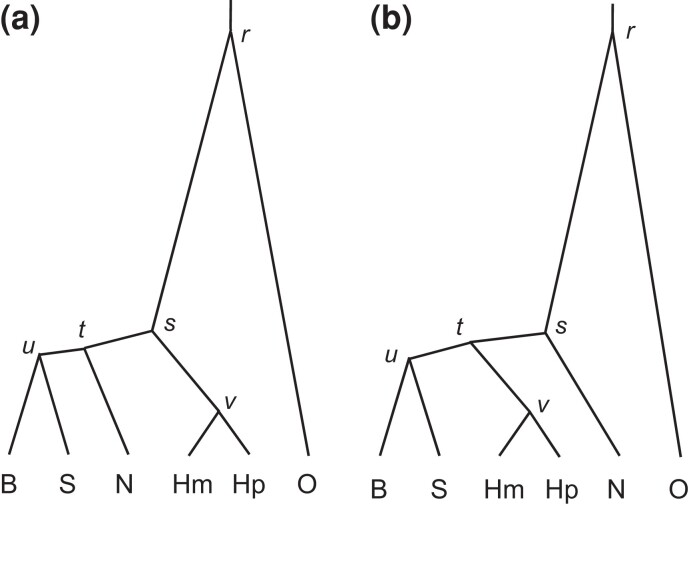
Species trees 1 and 2 for five species of gibbons: *Hylobates moloch* (Hm), *H. pileatus* (Hp), *Nomascus leucogenys* (N), *Hoolock leuconedys* (B), and *Symphalangus syndactylus* (S), with the human as the outgroup (O). These are the top two species trees in the species-tree analysis of genomic data by Shi and Yang (2018) (the A01 analysis of Yang 2015). Branches are drawn to represent the posterior means of divergence times (τs) in the bpp analysis of the noncoding data under the JC+clock model.

**Fig. 8.**
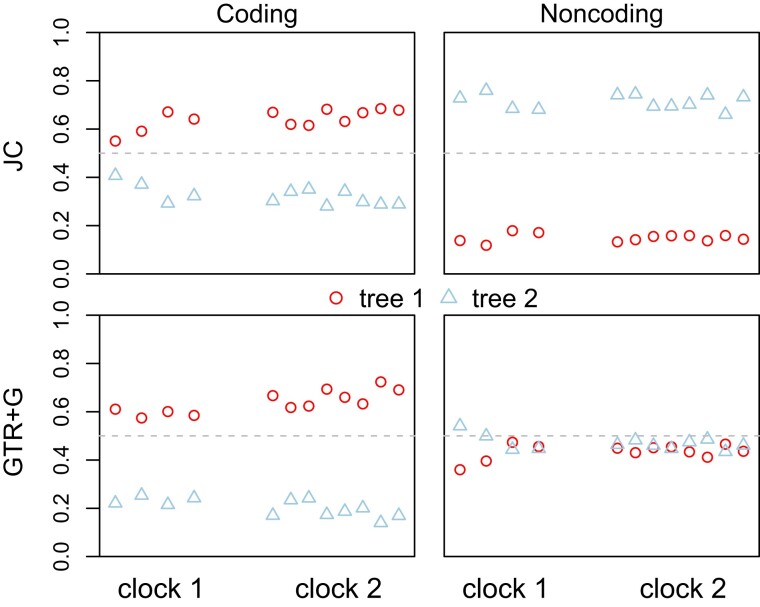
Posterior probabilities for species trees 1 and 2 for the gibbons (fig. 7) obtained from bpp analysis of the coding and noncoding datasets under different clock models. In each panel are presented two replicate runs for each of six analyses, specified as (1) clock = 1 (strict clock, one rate for all loci); (2) locusrate = 1 0 0 5 iid, clock = 1 (strict clock, i.i.d. rates $\mu_{i}$ among loci); (3) locusrate = 1 0 0 5 iid, clock = 2 10 100 5 iid LN (clock 2, i.i.d. prior for $\mu_{i}$ and $\nu_{i}$ among loci, and log-normal kernel); (4) locusrate = 1 0 0 5 iid, clock = 2 10 100 5 iid G (clock 2, i.i.d. prior for $\mu_{i}$ and $\nu_{i}$ among loci, and gamma kernel); (5) locusrate = 1 0 0 5 dir, clock = 2 10 100 5 dir LN (clock 2, dir prior for $\mu_{i}$ and $\nu_{i}$ among loci, and log-normal kernel); (6) locusrate = 1 0 0 5 dir, clock = 2 10 100 5 dir G (clock 2, dir prior for $\mu_{i}$ and $\nu_{i}$ among loci, and gamma kernel). The strict clock (clock 1) is assumed in the first two analyses while the independent-rates model (clock 2) is assumed in the next four analyses. The substitution model is either JC or GTR+Γ. Inverse-gamma priors are assigned on τ and θ.

We analyzed two datasets from five species of gibbons, with the human used as the outgroup (fig. 7). The datasets consist of 500 noncoding loci and 1000 coding loci, respectively, and were analyzed previously under the strict clock and the JC model (fig. 3*A*&*B* in Shi and Yang 2018). Here we used the strict clock, either with or without locus-rate variation, and the independent-rates model (clock 2) with different distributions of overall rates ($\mu_{i}$) and rate variance parameters ($\nu_{i}$) among loci (iid vs. dir) and different distributions of branch rates for each locus (LN vs. G), with 4=2×2 prior settings. Both JC and GTR+Γ were used. The results are summarized in figure 8.

The strict clock (clock 1) and the independent-rates models (clock 2) produced very similar results in both datasets (fig. 8). For the coding dataset, all analyses, under both JC and GTR+Γ and under both clock 1 and clock 2, favored tree 1, with posterior ∼0.6, whereas tree 2 had ∼0.2 (fig. 8). For the noncoding dataset, tree 2 was the MAP tree with posterior ∼0.53 under JC while tree 1 had 0.20, as in Shi and Yang (2018). Under GTR+Γ, trees 1 and 2 received nearly equal support. As the substitution model had some impact on the posterior probabilities of species trees in one of the two datasets, we conducted the same analysis using each of the 35 blocks of loci from the gibbon genome of Shi and Yang (2018, fig. 3*A*&*B*), with the results summarized in supplementary figure S8, Supplementary Material online. Each block was analyzed under the JC or GTR+$\Gamma_{4}$ models and the strict clock (clock 1). The results for JC are nearly identical to those in Shi and Yang (2018, fig. 3*A*&*B*). Overall, the two mutation models produced highly similar results, no more different than in the simulated datasets of supplementary figure S7, Supplementary Material online.

**Fig. 9.**
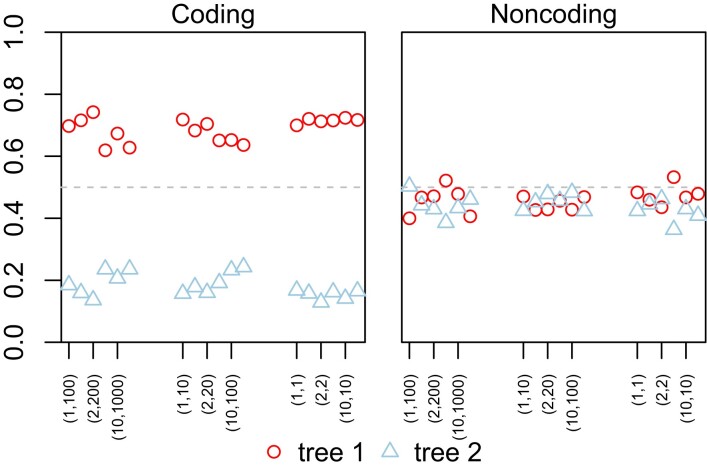
Posterior probabilities for species trees 1 and 2 (fig. 7) for the gibbon datasets under different priors in the relaxed-clock models. Two replicate runs are presented for each of nine priors, specified by locusrate = 1 0 0 5 iid, clock = 2 $\alpha_{\bar{\nu }}\;\beta_{\bar{\nu }}$ 5 iid G, where the parameters are given as ($\alpha_{\bar{\nu }}$, $\beta_{\bar{\nu }}$) = (1, 100), (2, 200), (10, 1000), (1, 10), (2, 20), (10, 100), (1, 1), (2, 2), and (10, 10). Note that the prior $\bar{\nu }∼G(\alpha_{\bar{\nu }},\beta_{\bar{\nu }})$ has the mean $\alpha_{\bar{\nu }}/\beta_{\bar{\nu }}$ and the variance $\alpha_{\bar{\nu }}/\beta_{\bar{\nu }}^{2}$. The GTR+Γ substitution model is assumed.

We evaluated the impact of different priors for the average rate variance parameter, $\bar{\nu }∼G(\alpha_{\bar{\nu }},\beta_{\bar{\nu }})$, on species tree estimation, assuming the GTR+Γ model. We used $\alpha_{\bar{\nu }}=1$, 2, 10 and $\beta_{\bar{\nu }}=\alpha_{\bar{\nu }},10\alpha_{\bar{\nu }},100\alpha_{\bar{\nu }}$, generating 3×3=9 priors in total. Note that the prior has mean $\alpha_{\bar{\nu }}/\beta_{\bar{\nu }}$ and variance $\alpha_{\bar{\nu }}/\beta_{\bar{\nu }}^{2}$, so that the mean reflects our prior assumption about the extent of clock violation while $\alpha_{\bar{\nu }}$ measures the confidence in the prior mean. The prior mean varied from 0.01 (very slight clock violation) to 1 (very severe clock violation). The different priors produced the same MAP trees with very similar posterior probabilities, suggesting that the analyses were robust to the priors (fig. 9).

#### Parameter Estimation under Different Clock Models and Priors

**Fig. 10.**
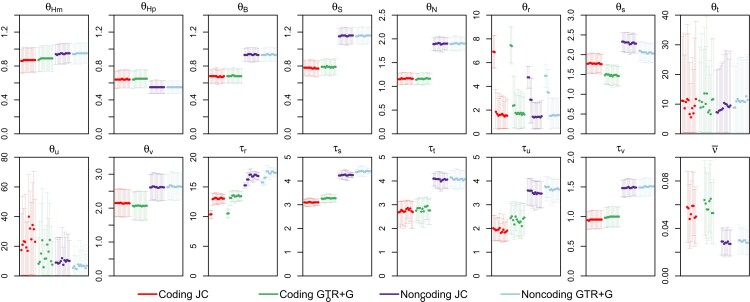
Posterior means and 95% HPD CIs for the 16 parameters in the MSC model on species tree 1 (fig. 7) in analyses of the gibbon datasets under different clock models. Estimates of θs and τs are multiplied by $10^{3}$. Each panel includes two replicate runs under six clock models as in figure 8, specified by (1) clock = 1 (strict clock, one rate for all loci); (2) locusrate = 1 0 0 5 iid, clock = 1 (strict clock, i.i.d. rates $\mu_{i}$ among loci); (3) locusrate = 1 0 0 5 iid, clock = 2 10 100 5 iid LN (clock 2, i.i.d. prior for $\mu_{i}$ and $\nu_{i}$ among loci, and log-normal kernel); (4) locusrate = 1 0 0 5 iid, clock = 2 10 100 5 iid G (clock 2, i.i.d. prior for $\mu_{i}$ and $\nu_{i}$ among loci, and gamma kernel); (5) locusrate = 1 0 0 5 dir, clock = 2 10 100 5 dir LN (clock 2, dir prior for $\mu_{i}$ and $\nu_{i}$ among loci, and log-normal kernel); (6) locusrate = 1 0 0 5 dir, clock = 2 10 100 5 dir G (clock 2, dir prior for $\mu_{i}$ and $\nu_{i}$ among loci, and gamma kernel). The panel for $\bar{\nu }$ shows two replicate runs for each of the four clock-2 analyses.

We then examined the estimates of parameters in the MSC model under the same six clock models as in figure 8, with results shown in figure 10. First, we note that species divergence times (τ) and population sizes (θ) for modern species were well estimated with narrow CIs, but population sizes for ancestral species were poorly estimated with wide CIs, especially for species corresponding to short internal branches in the tree.

Second, parameter estimates were overall very similar between the mutation models (JC and GTR+Γ) and between the strict clock (clock 1) and relaxed-clock (clock 2) models (fig. 10). One exception was the impact of the locus-rate variation on estimation of species divergence time and population size for the root population on the species tree ($\tau_{r}$ and $\theta_{r}$ in fig. 7). Ignoring mutation rate variation among loci is known to lead to overestimation of the ancestral population size and underestimation of the species divergence time. This effect was noted by Burgess and Yang (2008) and affects mostly the root of the species tree only. For those data, the locus-rate variation had a slightly larger effect than the clock models.

Third, estimates of the rate variance parameter were overall small ($\bar{\nu }<0.1$) and had large uncertainties, consistent with our expectation that the clock holds approximately for those data as the species are closely related. The large uncertainties in $\bar{\nu }$ may be due to the small species tree with only five species. We note that in this case estimates of $\bar{\nu }$ were similar between the gamma and log-normal models, even though the parameter has different interpretations in the two models.

Finally, estimates of τs and θs were smaller for the coding data than for the noncoding data. This is because the neutral mutation rate is reduced in the coding loci by purifying selection removing deleterious nonsynonymous mutations. Indeed Shi and Yang (2018) found that the posterior means under the JC+clock model were nearly perfectly linear between the two sets of data, with the regressions $\tau_{\left(C\right)}=0.73\tau_{\left(\mathrm{NC}\right)}$ and $\theta_{\left(C\right)}=0.62\theta_{\left(\mathrm{NC}\right)}$. Since our estimates under clock 2 and GTR+Γ were nearly identical to those under JC+clock, the same relationships apply to the estimates here.

**Fig. 11.**
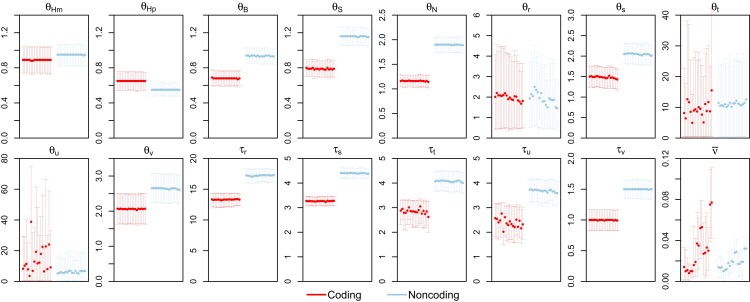
Posterior means and 95% HPD CIs for 16 parameter in the MSC model on species tree 1 for the gibbons (fig. 7) using different priors. The prior is specified as follows, as in figure 9: locusrate = 1 0 0 5 iid, clock = 2 $\alpha_{\bar{\nu }}\;\beta_{\bar{\nu }}$ 5 iid G, with $\left(\alpha_{\bar{\nu }},\beta_{\bar{\nu }})=(1,\;100\right)$, (2, 200), (10, 1000), (1, 10), (2, 20), (10, 100), (1, 1), (2, 2), and (10, 10). Estimates of τ and θ are multiplied by $10^{3}$.

We then evaluated the impact of the different priors for the rate variance parameter ($\bar{\nu }$) on parameter estimation, with the GTR+Γ model assumed (fig. 11). The different priors had virtually no impact on the species divergence times (τ) and population sizes (θ) for modern species, parameters that were well estimated, but had some minor effects on the ancestral population sizes, which were poorly estimated. However, estimates of the variance parameter $\bar{\nu }$ were affected by the prior (fig. 11). The posterior mean of $\bar{\nu }$ and the CI width increased with the increase in the prior mean, $\alpha_{\bar{\nu }}/\beta_{\bar{\nu }}$, and the prior mean had more impact than the prior variance. The sensitivity of $\bar{\nu }$ estimates to the prior (fig. 11) and the large CIs (figs. 10 and 11) both reflect the low information content about the parameter in the data.

In sum, our bpp analyses of the gibbon datasets (figs. 8–11) confirmed the expectation that JC+clock is adequate for shallow species trees when the species are closely related, the molecular clock approximately holds, and the sequences in the alignments are highly similar. JC is clearly an extremely unrealistic model (for example, the frequencies of the four nucleotides are rarely ∼0.25 each), but because the role of the mutation model in bpp is mainly to correct for multiple hits, the realism of the mutation model used is unimportant when the sequences are highly similar. We recommend the use of JC+clock for analysis of genomic data for closely related species, as it is far more efficient computationally than GTR+Γ.

### Analysis of the Ratite Data

We used the independent-rates model (clock 2) to analyze a dataset of 250 UCE loci to infer the species tree for the flightless birds (Palaeognathae) (fig. 5). Four clock models were used with either the iid or dir distributions of overall rates ($\mu_{i}$) and variances ($\nu_{i}$) among loci, and with either the log-normal (LN) or gamma (G) distributions of branch rates. Preliminary runs suggested that several clades had total support (with posterior ∼1), irrespective of the model and prior. We thus applied four clades or topology constraints to reduce the space of MCMC species-tree search: the kiwis (four species), tinamous (four species), rheas (two species), and emu+cassowary (two species), besides using the ostrich as the outgroup (fig. 5). We ran each of the four analyses 40 times, with four different starting species trees. The MAP trees and posterior probabilities are shown in supplementary figure S9, Supplementary Material online. The MCMC algorithm for stochastic tree search showed serious mixing problems, as seen by the differences among the replicate runs. The starting trees did not have an impact in this case. Tree 2 was the MAP tree in more replicate runs than any other tree under all four models (it was the MAP tree in 48.1% of the 4×40 runs) (supplementary fig. S9, Supplementary Material online). We suggest that the MAP tree was tree 2 under all four models, and combined samples across replicate runs in which the MAP tree was tree 2 to calculate the posterior probability for tree 2 to be 0.90, 0.87, 0.92, 0.85, for the four models. We discuss MCMC mixing problems later in the Discussion section.

In tree 2 (fig. 5), the rheas diverged first, followed by the divergence of the tinamou+moa clade from the kiwi-emu clade. Cloutier et al. (2019) analyzed the full UCE data of 3158 loci and used both ostrich and chicken as the outgroups, recovering tree 1 (fig. 5) as the estimate using both astral and MP-est, which has the tinamou+moa clade diverging first. A number of factors might explain the difference, including data filtering, the use of all or a subset of the loci, the different outgroups, and the different methods (bpp vs. summary methods).

To identify the possible reasons, we applied our filters to all three types of noncoding nuclear markers from Cloutier et al. (2019): the UCEs (ultraconserved elements), the introns, and the CNEEs (conserved nonexonic elements). For each filtered dataset, we used astral to infer the species tree with different subsets of loci, with three outgroup options: (i) the chicken and the ostrich, (ii) the ostrich only, and (iii) the chicken only. The results are summarized in supplementary table S1, Supplementary Material online. With the ostrich as the outgroup, the astral analysis of the 250-loci UCE data produced tree 2 as the estimate (supplementary table S1, Supplementary Material online), consistent with the bpp analysis. However, tree 1 was recovered when the chicken was used as the outgroup. With the ostrich+chicken outgroup, astral analyses of the full UCE and introns data recovered tree 2 as the estimate, while Cloutier et al. (2019) recovered tree 1; this difference should be due to our filtering of the data (supplementary table S1, Supplementary Material online). In sum, data filtering and the different outgroups had major impacts on species tree estimation in the ratite datasets. We note that Simmons et al. (2022) found similar dependence of the astral and MP-est results on the use of the outgroup species and argued that the chicken may not be the best outgroup species for rooting the ratite tree.

In the analysis of the same 250-loci UCE dataset, both bpp and astral produced tree 2 as the estimate, but the posterior probabilities for tree 2 from bpp were much higher than the local node support values from astral, that is, 0.93 for N1 and 0.67 for N2 in tree 2 (fig. 5, supplementary table S1, Supplementary Material online). This may be due to the fact that astral uses reconstructed gene tree topologies as data and ignores information in gene-tree branch lengths whereas bpp makes use of both sources of information, potentially increasing power. However, the two measures of support may not be directly comparable.

**Fig. 12.**
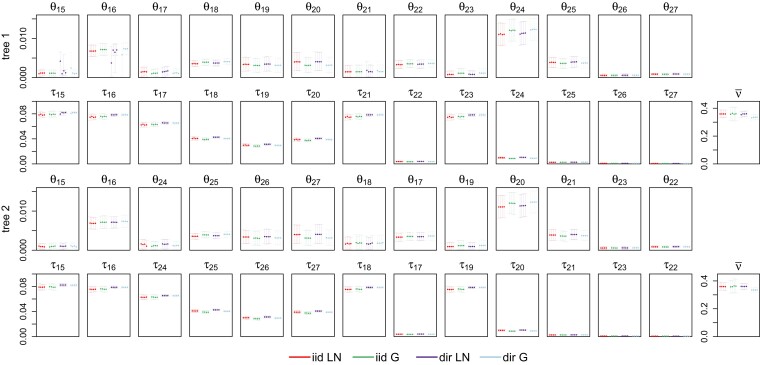
Posterior means and 95% HPD CIs for the 27 parameters in the MSC model on species tree 1 (fig. 5) in analyses of the ratite dataset under different clock models. The independent-rates model (clock 2) was assumed, with four prior settings concerning the distribution of overall rates ($\mu_{i}$) and rate variance parameters ($\nu_{i}$) among loci (iid vs. dir) and concerning the distribution of the branch rates (G vs. LN). Each panel shows four replicate runs for each of the four clock model settings.

Next we ran bpp to estimate the parameters under the MSC+relaxed clock model with the species tree fixed. Clock 2 (independent rates) was used together with GTR+Γ. This is the A00 analysis (Yang 2015), which did not suffer from serious mixing problems as in the A01 analysis. The posterior means and 95% HPD CIs for all parameters for species trees 1 and 2 of figure 5 are shown in figure 12. The CIs for most parameters were narrower than those from the simulated data (fig. 6), suggesting that the real dataset was more informative than the simulated datasets, presumably due to the fact that the average sequence length among the 250 UCE loci is 2525, much greater than the sequence length used in the simulation (500 sites).

## Discussion

### Simulation of Gene Trees and Sequence Alignments under the MSC+relaxed Clock Model

We have implemented a simulation procedure to generate gene trees with branch lengths and sequence alignments at multiple loci under the MSC+relaxed clock model. The simulation follows the model formulation of figure 3 (see the Materials and Methods section) and can adopt the GTR+Γ substitution model (Yang 1994a, 1994b) or its special cases, with the substitution parameters such as base frequencies or the gamma shape parameter for rate variation among sites sampled randomly among loci. We generate gene trees (topologies and coalescent times) on a rooted species tree with node ages representing species divergences times, and then use a rate-evolution model to simulate the substitution rates for different branches at different loci. The species divergence times, coalescent times and branch rates determine the branch lengths on the gene trees (fig. 1*a*), which can be used to generate the sequence alignment for the locus. Parameters in this MSC+relaxed clock model include species divergence times (τs), population sizes for both modern and ancestral species (θs), and parameters in the rate-evolution model (e.g., $\mu_{i}$ and $\nu_{i}$). The species tree is always rooted and ultrametric, whereas the gene trees are rooted but not ultrametric, with the branch lengths given by the products of time duration and species-specific and locus-specific substitution rates (fig. 1*a*). The asymptotic performance of the inference method is then assessed by letting the number of loci approach infinity while the sequence length is finite and fixed.

Recently, Roch et al. (2019) studied the inconsistency of coalescent-based summary methods, as well as partitioned and unpartitioned concatenation methods, for species tree estimation when the molecular clock is violated. An unrooted gene tree for four taxa was used to generate sequence data at multiple loci, which had two long external branches (with length ρ, measured in the probability of different sites) on two sides of the short internal branch, while the other three branches (one internal and two external) had the length $\rho^{3}$. This is the characteristic long-branch attraction (LBA) tree studied by Felsenstein (1978). When ρ is small and the sequence length is fixed, all summary methods of species tree estimation were found to be inconsistent, converging to an incorrect species tree when the number of loci or the number of gene trees approach ∞. Even though the maximum-likelihood (ML) method is consistent in recovering the gene trees (when the number of sites in the sequence approaches ∞, at a fixed finite sequence length it may recover a wrong gene tree (the LBA tree) with a higher probability than the true gene tree. As a result the more probable incorrectly reconstructed gene tree becomes a (statistically inconsistent) estimate of the species tree, when the number of loci approaches ∞. The result is interesting and highlights the importance of accounting for gene-tree reconstruction errors in species tree estimation. Nevertheless, the framework adopted by Roch et al. (2019) for evaluating the statistical properties of a species tree estimation method does not appear to be valid. The gene trees considered by Roch et al. (2019) vary in branch lengths in only one dimension, and are akin to isolated datasets which in total have near-zero probability of occurrence under an MSC model with violated clocks. One cannot draw valid statistical conclusions about the inference method based on such isolated datasets. Correctly gene trees with branch lengths are random variables, and both the gene-tree topology and all its five branch lengths should vary, as specified by the MSC and the rate-change model.

The simulation procedure implemented in bpp may provide a flexible tool for generating multilocus sequence datasets under the MSC with relaxed clocks and realistic substitution models, useful for studying the statistical performance of methods for estimating the species tree and divergence times.

### Mixing Issues of the MCMC Algorithm in bpp

Our comprehensive tests suggest that our implementation of the relaxed clock models (clock 2 and clock 3) are correct in that the MCMC samples from the posterior under the model. However, we observed MCMC mixing issues in the algorithm for changing species tree, in particular under the correlated-rates model (clock 3). Mixing is considerably poorer under the relaxed-clock models than under the strict clock. The main reason appears to be the increased dimension in the trans-model move. Note that species trees correspond to different statistical models, while τs, θs, and the locus-specific rate variance parameters and branch rates ($\mu_{i},\nu_{i},r_{ij}$) may all be considered parameters in the model. When we change a species tree through an NNI or SPR move (Yang and Rannala 2014; Rannala and Yang 2017), we modify the gene trees at the multiple loci to avoid conflicts, and the branch rates ($r_{ij}$) are transferred to the new trees at each locus, necessitating the re-evaluation of the sequence likelihood. The branch rates $r_{ij}$ did not exist under the clock, and their introduction in the relaxed-clock models increases the dimension of the MCMC algorithm considerably, leading to much reduced acceptance rate of the species-tree proposal. Similarly, the SPR move is “larger” under the correlated-rates model (clock 3) than under the independent-rates model (clock 2), involving changes to more variables, which may explain why clock 3 had even more severe mixing problems. While we were able to run bpp under the strict clock on datasets with >10,000 loci (Rannala and Yang 2017; Shi and Yang 2018), here we encountered mixing problems with species tree estimation with hundreds or even dozens of loci.

It may be noted that mixing under the relaxed-clock models was better for the two gibbon datasets with 500 or 1000 loci than for the ratite dataset with only 250 loci. This may be because there are more sequences per locus in the ratite dataset and furthermore the ratite sequences are more divergent so that each ratite locus is more informative than a gibbon locus. In large or informative datasets, the within-model parameter posterior becomes sharper, making it harder to move across models as the proposed parameters are likely to miss the spike in the parameter posterior under the new model.

Thus, our implementation in bpp of the MSC+relaxed clock model for species tree estimation (Yang 2015, the A01 analysis) is currently only feasible for use with small datasets, and should be considered a proof of concept. We leave it to future work to improve the mixing properties of the algorithm, so that the models can be applied to datasets with thousands of loci. We note that the prior on θ may affect MCMC mixing, and in particular the gamma and inverse-gamma priors have different features related to mixing. First, heavy-tailed priors on θ may cause mixing problems because they sometimes generate implausibly large θs for populations represented by short internal branches on the species tree (e.g., $\theta_{t}$ and $\theta_{u}$ in the gibbon trees in fig. 10), possibly because extremely large ancestral θs may make an implausible species tree look reasonable. The gamma is a light-tailed distribution while the inverse-gamma is heavy-tailed. Second, integrating out θs analytically reduces the dimension of the MCMC algorithm and helps mixing. The inverse-gamma is a conjugate prior for θs and allows θs to be integrated out analytically, while the gamma does not. Whether the gamma or the inverse-gamma is a better prior may thus depend on the particular datasets.

One idea worth exploring is to discretize the branch rates as an approximation to the continuous rates generated in the rate-evolution process and then to sum over the discrete rates analytically in the pruning algorithm, as achieved in the so-called speed-dating algorithm (Akerborg et al. 2008). If the branch rates are integrated out, they will not contribute to the dimension of the problem in the cross-tree proposal. Another idea is to simultaneously change the branch rates and coalescent times for each locus such that the gene-tree branch lengths stay fixed. This was originally proposed by Thorne et al. (1998) in the phylogenetic dating context and recently implemented in the context of MSC with relaxed clocks by Douglas et al. (2022) in StarBeast3. We note that recent algorithmic improvements in StarBeast3 have made the program feasible for datasets as large as 100 loci (Douglas et al. 2022, table 3).

### Assumptions and Utility of the Current Algorithms

Our algorithm for parameter estimation when the species tree is fixed (the A00 analysis, Yang 2015) does not seem to suffer from the mixing problems mentioned above. While the GTR+Γ model involve much more computation than the JC model, proposals changing the parameters in GTR+Γ at different loci are parallelized. We suggest that the current implementation in bpp may be most useful for estimating important population parameters (such as species divergence times, population sizes, and even the magnitude of rate variation over time), after the species tree topology is estimated using computationally efficient two-step methods such as astral or MP-est.

We also envisage examining the posterior distribution of gene trees at individual loci to identify genes that show unusual phylogenetic relationships as a possible indication for natural selection. The posterior distribution of substitution rates between loci might be used to identify genes that are co-evolving, for example, with strongly correlated branch rates. We expect such analysis to have power only if large species trees with many species are analyzed.

Here we examine some of the assumptions made in the MSC-relaxed clock models. First the models implemented here ignore cross-species introgression or migration. Ignoring gene flow when it exists may cause serious underestimation of species divergence times, as the model of no gene flow will then misinterpret the reduced sequence divergences between species due to gene flow as evidence for recent species divergence. We have recently implemented the multispecies-coalescent-with-introgression (MSci) model in bpp assuming the strict clock model (Flouri et al. 2020). It will be straightforward to extend the model to work under the relaxed clocks.

Similarly both the independent- and correlated-rates models may be unrealistic for some species groups. One assumption made by all current relaxed-clock models is that substitution rates evolve independently among loci, whereas there exists evidence for strong lineage effects in substitution rates, in that almost all genes from a fast-evolving lineage tend to have high rates (Lee and Ho 2016; Xu and Yang 2016). For example, in mammals, rodents tend to have high rates than primates, and the effect is correlated with life-history traits of the species which affect all genes in the genome (Li et al. 1987; Amster and Sella 2016). We leave it to future work to implement such models of rate evolution with lineage effects or correlated rate evolution among loci. Models of independent rate evolution that ignore the correlation among loci are still able to fit arbitrary rates to branches on the gene trees, but may be expected to exaggerate the amount of information in the data. In other words, under relaxed-clock models accommodating lineage effects of rate evolution, the lineage rates will be confounded with species divergence times, making relaxed-clock dating extremely challenging (Yang and Donoghue 2016). Under the independent-rates model, the infinite- and finite-sites theories (Rannala and Yang 2007; Zhu and Yang 2015) predict that when the number of loci increases, the precision of species divergence times will approach a fixed limit given under the strict-clock model, reflecting the uncertainties in the fossil calibrations. Strong lineage effects in rate evolution may change the asymptotics of relaxed-clock dating, and in particular, the prior of divergence times specified by the model of cladogenesis is expected to have a significance impact on the posterior of divergence times (Xu and Yang 2016).

## Materials and Methods

### Simulation to Evaluate Species Tree Estimation

We conducted two sets of simulations to evaluate the performance of the relaxed clock models implemented in bpp for species tree estimation and parameter estimation, respectively. In each, data of sequence alignments at multiple loci were simulated using the simulate option of bpp4. Simulation consisted of three steps: (i) generation of gene trees and coalescent times for each locus under the MSC model, (ii) simulation of substitution rates for each locus along species-tree branches (which determine gene-tree branch lengths), and (iii) simulation of sequences along branches of the gene trees. The resulting sequences at the tips of the gene trees constitute the data. A sample bpp control file (MCcoal.ctl) used for the simulation is shown in supplementary figure S10, Supplementary Material online.

The first set of simulations used the four-species tree (A,B,C, and outgroup O) of figure 4 with an independent-rates model (clock 2). The simulated sequences were analyzed using bpp under all three clock models, and using two summary methods, astral (Mirarab and Warnow 2015) and MP-est (Liu et al. 2010). The species tree had divergence times $\tau_{R}=0.2$, $\tau_{S}=0.105$, and $\tau_{T}=0.1$, and population size parameters $\theta_{R}=\theta_{S}=0.01$, $\theta_{T}=0.05$ (fig. 4). The short internal branch, in $2(\tau_{S}-\tau_{T})/\theta_{T}=0.2$ coalescent units, makes the species tree challenging to recover. We sampled one sequence per species per locus.

Overall rates ($\mu_{i}$) among loci were either constant or variable, and in each case, the same model is used in both simulation and analysis of the data. With variable rates, $\mu_{i}$ for locus i was sampled from a gamma distribution G$\left(\alpha_{\mu },\alpha_{\mu }\right)$ with $\alpha_{\mu }=5$ (locusrate = 1 5 iid). Given $\bar{\nu }$ (either 0.01 or 0.1), the rate variance parameter $\nu_{i}$ for locus i was generated from G$\left(\alpha_{\nu },\alpha_{\nu }/\bar{\nu }\right)$ with $\alpha_{\nu }=5$ (with the specification clock = 2 0.1 5 iid g in the case of $\bar{\nu }=0.1$, for example). Given the overall rate $\mu_{i}$ and the variance parameter $\nu_{i}$ for each locus i, the rate $r_{ij}$ for (species-tree) branch j at locus i was sampled from the gamma distribution with mean $\mu_{i}$ and variance $\nu_{i}$ (eq. 9). A branch length on a gene tree is specified as the sum of branch segments corresponding to populations that the branch traverses (see fig. 1*a*).

Sequences were simulated under a GTR+$\Gamma_{5}$ substitution model (Yang 1994a, 1994b), with the parameters in the model varying among loci. For each locus, the base frequencies $\pi =(\pi_{T},\pi_{C},\pi_{A},\pi_{G})$ were generated from a Dirichlet distribution π∼ Dir($\alpha_{T},\alpha_{C},\alpha_{A},\alpha_{G}$) with parameters $\left(\alpha_{T},\alpha_{C},\alpha_{A},\alpha_{G})=(10,10,10,10\right)$. The exchangeability parameters for the GTR model (Yang 1994a) were also generated from a Dirichlet distribution $q=(a,b,c,d,e,f)∼\mathrm{Dir}(\alpha_{a},\alpha_{b},\alpha_{c},\alpha_{d},\alpha_{e},\alpha_{f})$ with parameters $\left(\alpha_{a},\alpha_{b},\alpha_{c},\alpha_{d},\alpha_{e},\alpha_{f})=(10,5,5,5,5,10\right)$; that is, the prior mean of the transition/transversion rate ratio (κ) is 2. The shape parameter for gamma distributed rates among sites at a locus was generated from G(2,2), with k=5 categories in the discrete-gamma model (Yang 1994b). Four values were used for the number of loci: L=10,20,100,200, with 500 sites per sequence and four sequences per locus. The number of simulated replicate datasets was 100. Using two rate variance ($\bar{\nu }$) values, two locus-rate variation models, four data sizes (L) and 100 replicates, we simulated a total of 2×2×4×100=1,600 datasets.

The simulated multilocus sequence datasets were analyzed to infer the species tree using bpp4 (Flouri et al. 2018) as well as astral (Mirarab and Warnow 2015) and MP-est (Liu et al. 2010). The outgroup was used to root the tree in both astral and MP-est. In both the astral and MP-est analyses, RAxML was used to infer the unrooted gene trees under the JC model and the most common gene tree was the species tree estimate. The bpp analysis used either sequences of only the three ingroup species, or sequences of all four species. In the latter case, O was used as the outgroup. Note that bpp always operates on rooted trees with node ages, so that rooted trees are inferred under relaxed-clock models whether or not outgroups are included in the data. We expect that use of an outgroup should provide additional information about the rooted species tree. We assign gamma priors on the age of the root, $\tau_{0}∼G(2,15)$ with mean 0.133, which is too small for the 3-species data and too large for the 4-species data. The population size parameters are assigned the gamma prior θ∼G(2,200) with mean 0.01. When analyzing sequences simulated with rate variation among loci, the locus-rate option was used in the bpp analysis (locusrate = 1 0 0 5 iid), with $\alpha_{\mu }=5$, so that the overall rates for loci have the i.i.d. prior $\mu_{i}∼G(\alpha_{\mu },\alpha_{\mu })$ with mean 1.

We used all three clock models to analyze the data: clock 1 (strict clock), clock 2 (independent rates), and clock 3 (correlated rates). We expect clock 1 to work best when $\bar{\nu }=0.01$ (slight clock violation) and worst when $\bar{\nu }=0.1$ (serious clock violation). For data simulated with $\bar{\nu }=0.01$ the clock 2 prior is specified as clock = 2 2 200 5 iid g, with $\alpha_{\bar{\nu }}=2,\beta_{\bar{\nu }}=200,\alpha_{\nu }=5$, so that $\bar{\nu }∼G(2,200)$ with prior mean 0.01, and the rate variance parameters for loci $\nu_{i}\;|\;\bar{\nu }∼G(5,5/\bar{\nu })$ (fig. 2). For data simulated with $\bar{\nu }=0.1$ the clock 2 prior was adjusted to clock = 2 2 20 5 iid g. In both cases the rates for branches were modelled using a gamma kernel. The prior for clock 3 was specified similarly to clock 2, using clock = 3 2 200 5 iid g with $\alpha_{\bar{\nu }}=2,\beta_{\bar{\nu }}=200,\alpha_{\nu }=5$ for data simulated with $\bar{\nu }=0.01$; and clock = 3 2 200 5 iid g with $\alpha_{\bar{\nu }}=2,\beta_{\bar{\nu }}=20,\alpha_{\nu }=5$ for data simulated with $\bar{\nu }=0.1$. The rates for branches were modeled using the bivariate log-normal density. The nucleotide substitution model assumed was either JC or GTR+$\Gamma_{5}$ (the true model). Uniform Dirichlet priors are used for the exchangeability parameters in the GTR model and for the stationary base frequencies.

With 1,600 datasets, 3 clocks, 2 substitution models, and 2 outgroup choices, we conducted a total of 1,600×3×2×2=19,200bpp analyses. A sample bpp control file is provided in supplementary figure S10*b*, Supplementary Material online. We conducted pilot runs to determine the length of the Markov chain needed for convergence. In the final setting, we used 32,000 iterations for burn-in, and then took $2\times 10^{5}$ samples, sampling every 2 iterations. Running time for each analysis ranged from ∼30 s for the small datasets of L=10 loci analyzed under the strict clock and JC without outgroup and without locus-rate variation to ∼15 h for the large datasets of L=200 loci analyzed under clock 2 and GTR+$\Gamma_{5}$ with locus rate variation and with outgroup.

### Simulation to Evaluate Parameter Estimation

The second set of simulations assessed the performance of parameter estimation under the MSC model when the clock is violated. We used parameter estimates for the ratites species tree of Cloutier et al. (2019) (species tree 1, fig. 5*a*) obtained from the bpp analysis of the 250 UCE loci to simulate datasets under the independent-rates model (using clock = 2 0.35 5 iid g, with $\bar{\nu }=0.35$). The species divergence times (τ) were estimated from the UCE data, with $\tau_{16}=0.0783$ for the root of the non-ostrich Palaeognathae clade and $\tau_{15}=0.0820$ for the separation of the ostrich (fig. 5*a*). For the population size parameters (θ), we used two values 0.001 and 0.005, and assigned the small value to six branches with small empirical estimates and the large value to the branches with large estimates. The GTR+$\Gamma_{5}$ model was used to simulate data, with parameters in the model sampled for every locus, as described above. We simulated 100 replicate datasets, each of 250 loci, with one sequence sampled per species and with the sequence length of 500 sites. The simulation control file is included as Supplementary material.

Each simulated dataset was analyzed using bpp4 to estimate the model parameters with the species tree fixed (the A00 analysis, Yang 2015). Both JC and GTR+Γ (the true model) were used in the analysis, assuming either the strict clock or clock 2 (i.e., clock = 2 2 5 5 iid g). With two substitution models and two clocks, there are in total 400 bpp analyses. In all runs rates were assumed to vary across loci (locusrate = 1 0 0 5 iid) (fig. 2). The age of the root was assigned the inverse-gamma prior $\tau_{15}∼\mathrm{invG}(3,0.2)$ with mean 0.2/(3−1)=0.1. Population sizes were assigned the prior θ∼invG(3,0.006) with mean 0.003. We used 32,000 iterations for burn-in, and then took $2\times 10^{5}$ samples. Running time was ∼7 h under the clock+JC model, ∼11 h under clock 2+JC, ∼41 h under clock 1 with GTR+Γ, and ∼154 h under clock 2 with GTR+Γ.

### Analysis of the Gibbon Datasets

We analyzed two datasets from the gibbon genomes (Shi and Yang 2018) using the relaxed clock models. The coding and noncoding genomic datasets were generated by Carbone et al. (2014) and Veeramah et al. (2015) for five gibbon species: *Hylobates moloch* (Hm), *Hylobates pileatus* (Hp), *Nomascus leucogenys* (N), *Hoolock leuconedys* (B), and *Symphalangus syndactylus* (S), plus an outgroup (human). There were 12,413 noncoding loci, each of 1,000 bp, and 11,323 coding loci, each of 200 bp, with 17 sequences per locus. Here we used the first 500 noncoding loci and the first 1000 coding loci, which correspond to block 1 in figure 3*A*&*B* of Shi and Yang (2018), who analyzed the data under the JC+clock model.

We used both the strict clock (clock 1) and the independent-rates model (clock 2) to estimate the species tree, assuming either the JC (Jukes and Cantor 1969) or GTR+$\Gamma_{4}$ (Yang 1994a, 1994b) substitution models. We assigned inverse-gamma priors $\tau_{0}∼\mathrm{IG}(3,0.03)$ with mean 0.015 for the age of the species-tree root, and θ∼IG(3,0.004) with mean 0.002 for the population sizes, allowing θ to be integrated out analytically. For the GTR+Γ model, a gamma prior is assigned on the shape parameter α for among-sites rate variation: α∼G(1,1). We conducted pilot runs to determine the MCMC settings for convergence. The final settings are 16,000 iterations for burn-in, followed by $8\times 10^{5}$ samples (or $4\times 10^{5}$ samples when the influence of priors was examined), with a sampling frequency of 2 iterations. Each analysis is run twice to confirm consistency between runs. Running time using one thread was ∼57 h under the clock+JC model or ∼13 days under clock 2 with GTR+Γ for the coding dataset. For the noncoding dataset, it was ∼34 h under JC or ∼10 days under GTR+Γ.

We then examined the impact of the different prior assumptions about the rate variance parameter $\bar{\nu }$. Running time was ∼6 days for the coding dataset and ∼5 days for the noncoding dataset.

The species tree analysis recovered trees 1 and 2 of figure 7 as the maximum *a posteriori* (MAP) tree, as in Shi and Yang (2018). We then fixed the species tree to estimate the parameters in the MSC model including species divergence times and population sizes under different models about the molecular clock (clock 1 and clock 2). We used 16,000 iterations for burn-in, then taking $2\times 10^{5}$ samples, sampling every 2 iterations. Running time using two threads was ∼6 h under clock+JC or 66 h under clock 2 with GTR+Γ for the coding dataset. For the noncoding dataset, it was ∼8 h under clock+JC or ∼38 h under clock 2 with GTR+Γ.

We used the GTR+Γ model to analyze the data under different priors on the rate variance parameter ($\bar{\nu }$) in clock 2 to evaluate the posterior sensitivity to the prior in the estimation of the species tree and parameters. The MCMC settings were the same as above. Running time using two threads was ∼44 h for the coding dataset and ∼34 h for the noncoding dataset.

### Analysis of the Ratite Dataset

We used a subset of 250 loci from the data of 3,158 UCEs from the flightless birds (Palaeognathae) analyzed by Cloutier et al. (2019). There are 13 species, including the extinct little bush moa (*Anomalopteryx didiformis*), plus the ostrich as the outgroup (Cloutier et al. 2019). We omitted the more distant outgroup, chicken (see fig. 5). Manual inspection suggested that alignments at some loci had poor quality. We thus applied the following filters to improve the data quality.

- Step 1: remove sequences with on average >40% differences from other sequences in the alignment.
- Step 2: remove columns with no states (all gaps).
- Step 3: remove sequences that have >50% missing data.
- Step 4: remove columns with no states (all gaps).
- Step 5: remove loci that comprise >50% columns with missing data.

The number of UCE loci (alignments) after filtering was 2,278. Most sequences removed in steps 1 and 3 were from white-throated tinamou. The sequence length ranged from 966 to 11,018 sites among loci, with the mean 2510. We used the first 250 loci, with mean sequence length of 2525.

We estimated the species tree under clock 2 with four different prior settings, with either the iid (conditional i.i.d.) or dir (gamma-Dirichlet) distributions for the overall rate ($\mu_{i}$) and variance parameter ($\nu_{i}$) among loci, and either the gamma (G) or the log-normal (LN) distributions for species-tree branch rates at each locus. A typical setting is locusrate = 1 0 0 5 iid and clock = 2 2 20 5 iid G, specifying the iid prior for $\mu_{i}$ and $\nu_{i}$, and the gamma distribution for the branch rates (fig. 2). In all four prior settings, the mean rate variance parameter $\bar{\nu }∼G(2,20)$ with mean 2/20=0.1, representing serious clock violation.

Gamma priors are assigned to the MSC parameters: $\tau_{0}∼G(2,20)$ with mean 2/20=0.1 for the age of the species-tree root, and θ∼G(2,2000) with mean 0.001 for the population sizes. We assumed the GTR+Γ substitution model, with the gamma shape parameter for the rate variation among sites (Yang 1994b) assigned a gamma prior, α∼G(2,1).

Preliminary runs suggested that several clades had complete support, with posterior ∼1, irrespective of the model and prior. They were defined as five clade constraints during the Bayesian species tree search, to reduce the search space. These were the kiwis (4 species), tinamous (4 species), rheas (2 species), and emu+cassowary (2 species), with the ostrich as the outgroup (meaning that all 13 ingroup species form a clade) (see fig. 5). We used 32,000 iterations for burn-in, then taking $10^{5}$ samples, sampling every 2 iterations. Each of the four prior settings was run 40 times, using four different starting trees (10 runs for each starting tree). Each run took ∼10 days using two threads. This analysis produced species tree 2 of figure 5 as the best estimate.

We then reran bpp with the species tree fixed to estimate the parameters of the MSC model, such as species divergence times, population sizes, and the rate variance parameter ($\bar{\nu }$). The same settings were used as above except that $2\times 10^{5}$ samples were taken. Running time using 4 threads was ∼7 days.

## Supplementary Material

msac161_Supplementary_Data

## Data Availability

Simulation and inference under the MSC+relaxed clock models are implemented in bpp Version 4 or later (Flouri et al. 2018), using the --cfile and --simulate switches of the program, respectively. The software is distributed at its github site (https://github.com/bpp/). The models and methods implemented include the independent-rates model (clock 2), the correlated-rates model (clock 3), the MCMC proposals for species-tree change under the MSC+relaxed clock models, the GTR+Γ substitution model and its special cases, as well as simulation of gene trees and sequence alignments under the MSC+relaxed clock models. Data files for the gibbon and ratite datasets and the bpp control files for simulating and analyzing data under the relaxed-clock models are archived at http://abacus.gene.ucl.ac.uk/ziheng/data.html.
