# Correction to: Bayesian Phylogenetic Inference using Relaxed-clocks and the Multispecies Coalescent

**DOI:** 10.1093/molbev/msac246

**Published:** 2022-12-06

This is a correction to: Tomáš Flouri, Jun Huang, Xiyun Jiao, Paschalia Kapli, Bruce Rannala, Ziheng Yang, Bayesian Phylogenetic Inference using Relaxed-clocks and the Multispecies Coalescent, *Molecular Biology and Evolution*, Volume 39, Issue 8, August 2022, msac161, https://doi.org/10.1093/molbev/msac161

We should mention that Ogilvie et al. (2017) implemented a multispecies coalescent model with relaxed clocks in StarBEAST2, which assigns branch rates to species-tree branches rather than to gene-tree branches, as discussed by Xu and Yang (2016). The model does not allow the branch rates to change freely at different loci. In the notation of our paper, Ogilvie et al. (2017) assigns a rate μi for locus i, but the branch rate r_i j_ for locus i and species-tree branch j is constant for all i for each j, with L+ (2s−1) rate parameters in total for a species tree with s species and data of L loci, compared with L+ (2s−1)×L rate parameters in the model implemented in BPP. In the formulation of Ogilvie et al. (2017), the species-tree branch rates are shared by all loci and are confounded with the species divergence times (τ in our paper). We thank Drs Alexei Drummond and Huw Ogilvie for clarifications of the model implemented in StarBEAST2 (Ogilvie et al. 2017). Ogilvie HA, Bouckaert RR, Drummond AJ. 2017. StarBEAST2 brings faster species tree inference and accurate estimates of substitution rates. *Mol Biol Evol* 34:2101-2114.

