# Supplementary material for: Bayesian Phylogenetic Inference using Relaxed-clocks and the Multispecies Coalescent
Source: Mol Biol Evol. 2022 Jul 30;39(8):msac161. doi: 10.1093/molbev/msac161 (PMC9366188; doi:10.1093/molbev/msac161)
Supplement: msac161_Supplementary_Data [file msac161_supplementary_data.zip › bppRelaxedClock-SI.pdf]

Supplemental Information

SI text. Simulations to validate the MCMC algorithms in BPP

We present two sets of tests to validate our implementation of the MCMC algorithms under MSC-relaxed clocks in BPP. In the first set, we ran BPP with the likelihood fixed at 1 to confirm BPP is sampling from the prior, which may be analytically tractable. In the second set, we simulate many replicate datasets, each simulated using parameter values sampled from the prior, to confirm that the posterior, averaged over the replicate datasets, matches the prior.

Running the MCMC algorithm without data

The species tree topology is fixed at $((A,B),C)$ (fig. 1a), corresponding to the A00 analysis of Yang (2015). A dataset of three loci, with 2 sequences per species per locus, was generated and used. The control variable `usedata = 0` in BPP instructs the program to read the sequence data and use the number of sequences, but ignore the sequence length or the sequence data during the algorithm, with the likelihood always fixed at 1.

The five θ parameters in the MSC model (fig. 1a) are assigned the gamma prior $\theta \sim G(2,200)$, with mean 0.01. We plotted three of them: $\theta_A, \theta_{AB}, \theta_{ABC}$. The root age on the species tree is assigned a gamma prior, $\tau_{ABC} \sim G(2,20)$, with mean 0.1. Given τ_{ABC}, the node age τ_{AB} is assumed to be uniform, $\tau_{AB}|\tau_{ABC} \sim \mathbb{U}(0,\tau_{ABC})$, so that τ_{AB} has the prior density

$$\begin{aligned} f(\tau_{AB}) &= \int_0^\infty f(\tau_{AB}|\tau_{ABC}) f(\tau_{ABC}) \cdot \mathbb{I}_{\tau_{AB}<\tau_{ABC}} \,\mathrm{d}\tau_{ABC} \\ &= \int_{\tau_{AB}}^\infty \frac{1}{\tau_{ABC}} \cdot \frac{\beta^\alpha}{\Gamma(\alpha)} \tau_{ABC}^{\alpha-1} \,\mathrm{e}^{-\beta\tau_{ABC}} \,\mathrm{d}\tau_{ABC} \\ &= \frac{\beta}{\alpha-1} \times \int_{\tau_{AB}}^\infty \frac{\beta^{\alpha-1}}{\Gamma(\alpha-1)} \tau_{ABC}^{\alpha-2} \,\mathrm{e}^{-\beta\tau_{ABC}} \,\mathrm{d}\tau_{ABC} \\ &= \tfrac{\beta}{\alpha-1} \left[1 - G(\tau_{AB}, \alpha-1, \beta)\right], \end{aligned} \tag{A1}$$

where $\alpha = 2$ and $\beta = 20$ are parameters in the prior $\tau_{ABC} \sim \mathrm{G}(2,20)$, and $G(x,\alpha-1,\beta)$ is the CDF for the gamma distribution $\mathrm{G}(\alpha-1,\beta)$. The mean of τ_{AB} is $\mathbb{E}(\mathbb{E}(\tau_{AB}|\tau_{ABC})) = \frac{1}{2}\mathbb{E}(\tau_{ABC}) = 0.05$.

The GTR+Γ_4 substitution model (Yang, 1994a,b) is assumed, with the exchangeability rates $(a,b,c,d,e,f$, scaled to sum to 1) assigned the Dirichlet prior $\mathrm{dir}(2,1,1,1,1,2)$, with prior expectation of 2 for the transition/transversion rate ratio. The base-frequency parameters are assigned a uniform Dirichlet prior, $(\pi_T,\pi_C,\pi_A,\pi_G) \sim \mathrm{dir}(1,1,1,1)$. The shape parameter α for variable rates among sites is assigned the gamma prior $\alpha \sim G(5,5)$ with mean 1.

We used both the independent-rates (clock 2) and correlated-rates (clock 3) models, and for each examined four model settings, with different prior distributions of the overall rate and variance parameters (μ_i and ν_i) among loci (conditional i.i.d. versus gamma-Dirichlet), and different kernel distributions of branch rates (gamma versus log-normal). We thus included all eight new relaxed-clock model settings implemented in BPP:

2a) `locusrate = 1 0 0 5 iid, clock = 2 10 100 5 iid LN`
2b) `locusrate = 1 0 0 5 iid, clock = 2 10 100 5 iid G`
2c) `locusrate = 1 0 0 5 dir, clock = 2 10 100 5 dir LN`
2d) `locusrate = 1 0 0 5 dir, clock = 2 10 100 5 dir G`

3a) `locusrate = 1 0 0 5 iid, clock = 3 10 100 5 iid LN`
3b) `locusrate = 1 0 0 5 iid, clock = 3 10 100 5 iid G`
3c) `locusrate = 1 0 0 5 dir, clock = 3 10 100 5 dir LN`
3d) `locusrate = 1 0 0 5 dir, clock = 3 10 100 5 dir G`

We used a burn-in of 32000 iterations, after which we took 10^5 samples, sampling every 100 iterations. There are thus eight runs of BPP, each requiring minimal computation.

We monitored 12 parameters in the MSC-relaxed clock model for the species tree $((A,B),C)$, for which the prior marginals are analytically available for comparison. These are θ_A, θ_{AB}, θ_{ABC}, τ_{ABC}, τ_{AB}, and the locus-specific substitution parameters in the GTR+Γ_4: the exchangeability rates a,b,c,d,e,f and the gamma shape parameter α. The results are presented in figures S1&S2, which showed perfect matches between the prior and the posterior.

Bayesian simulation

A Bayesian simulation was performed under the MSC+relaxed clock model and GTR+Γ_4 in which the parameters used to generate each replicate dataset were simulated from the prior. The objective was to confirm that the posterior samples of parameters generated by applying BPP to the simulated replicate datasets, combined across replicate datasets, represent a sample from the prior (see eq. 17). Errors in the calculation of the priors or likelihood, or in the MCMC algorithms or in their implementations, are all likely to cause a mismatch. This is thus a more stringent test than simply running the MCMC without data and comparing against the prior as that approach does not involve calculation of the likelihood function.

We considered the same eight relaxed-clock model settings as in the first test. For each setting we generated $R = 200$ replicate datasets, with $8 \times 200 = 1600$ datasets in total. Each replicate dataset consisted of ten loci, with 2 sequences per species per locus and with the sequence length to be 500 sites. For each setting, we used the following two steps to simulate and analyze each replicate dataset:

1. Generate a simulation control file `MCcoal.ctl` by sampling parameters from the prior. The species

tree $((A,B),C)$ is fixed and θs and τs are sampled from their priors. Sample $\theta_i \sim G(2,200)$, $\tau_{ABC} \sim G(2,20)$ and $\tau_{AB} \sim \mathbb{U}(0, \tau_{ABC})$ (Yang and Rannala, 2010, eq. 2). Given the MSC parameters (θs and τs), the gene tree topologies and coalescent times are simulated under the MSC model (Rannala and Yang, 2003). The average overall rate $\bar{\mu}$ is fixed at 1 and, given $\bar{\mu}$, the overall rates for loci (μ_i) are simulated using either a uniform Dirichlet (`dir`) or a gamma distribution (`iid`). The average rate-variance parameter $\bar{\nu}$ is simulated from a gamma prior $G(10,100)$ and, given $\bar{\nu}$, the variance parameters for loci (ν_i) are simulated using either a uniform Dirichlet (`dir`) or a gamma distribution (`iid`). The rate-evolution model then allows simulation of branch rates for loci and specifies gene tree branch lengths for each locus (the sum of segment lengths for each branch) (fig. 1a). GTR exchangeability rates (a,b,c,d,e,f) are simulated for each locus from a Dirichlet distribution dir$(2,1,1,1,1,2)$, with prior expectation of 2 for the transition/transversion rate ratio. The base-frequency parameters are simulated from a uniform Dirichlet, $(\pi_T, \pi_C, \pi_A, \pi_G) \sim$ dir$(1,1,1,1)$. The shape parameter α for variable rates among sites is simulated as $\alpha \sim G(5,5)$. Given the gene tree topology and branch lengths, and the parameters in the GTR+Γ_4 model, we evolve the sequences along branches of the gene tree, and the sequences at the tips constitute the sequence data. The `simulate` option of BPP is used to generate the gene trees and sequence data. This step is also used to estimate the prior distributions for all parameters, even though the priors for some are analytically tractable (see below).

2. Create the inference control file and analyze the replicate dataset to sample from the posterior. The BPP control file for the A00 analysis (Yang, 2015) is the same for all replicate datasets. We used 32000 MCMC iterations as the burn-in, and then take 10000 samples, sampling every 50 iterations. This excessive 'thinning' is done to avoid very large files. Analysis of each replicate dataset took ~ 5 minutes.

The MCMC samples for the 200 replicates are merged and used to estimate the density using kernel density smoothing. We monitored 16 parameters or gene-tree features: $\theta_A, \theta_{AB}, \theta_{ABC}, \tau_{ABC}, \tau_{AB}$ on the species tree, and locus-specific variables: μ_1 and ν_1, GTR rates a,b,c,d,e,f and α, and tree height (TH or the root age) and tree length (TL or the sum of branch lengths) on the gene tree. The branch length on the gene tree is calculated as a sum over the different segments and is a function of species divergence times (τs), coalescent times (ts), and branch rates for the locus (r_{ij}) (fig. 1a). Note that the loci are exchangeable so we can average over the loci to get more precise estimates.

The empirical prior distributions generated during step 1 and the average empirical posterior distributions generated during step 2 are plotted in figures S3&S4. These show nearly perfect matches, as expected from theory (see eq. 17). Note that the priors for the 12 parameters examined in the first test above are analytically tractable, even though here we estimated them empirically using sampled values. The other four parameters (μ_1, ν_1, TH and TL) are locus-specific. The average overall rate $\bar{\mu}$ is fixed at 1. Given $\bar{\mu}$, the overall rate for the locus (μ_1) is generated using either the uniform Dirichlet distribution (`dir`) or the gamma distribution (`iid`). Thus μ_1 should have the mean $\mathbb{E}(\mu_1|\bar{\mu}) = \bar{\mu} = 1$. The average rate variance parameter $\bar{\nu}$ is sampled from the gamma prior $G(10,100)$. Given $\bar{\nu}$, the variance parameter for the locus (ν_1) is generated using either the uniform Dirichlet distribution (`dir`) or the gamma distribution (`iid`). Thus ν_1 should have the mean $\mathbb{E}(\nu_1) = \mathbb{E}(\mathbb{E}(\nu_1|\bar{\nu})) = 0.1$. We did not attempt to derive the prior distributions of μ_1 or ν_1 analytically. The gene tree topology, the coalescent times, and the branch rates for the locus are generated under the MSC+relaxed clock model (fig. 3). Branch lengths on the gene tree are then calculated by summing up the lengths of the segments (fig. 1a). Those gene-tree features do not appear to be tractable analytically. We plotted the gene-tree tree height (the age of the gene-tree root) and the tree length (the sum of branch lengths) and observed near-perfect matches (figs. S3&S4).

References

Cloutier, A., Sackton, T. B., Grayson, P., Clamp, M., Baker, A. J., and Edwards, S. V. 2019. Whole-genome analyses resolve the phylogeny of flightless birds (palaeognathae) in the presence of an empirical anomaly zone. *Syst. Biol.*, 68(6): 937–955.

Rannala, B. and Yang, Z. 2003. Bayes estimation of species divergence times and ancestral population sizes using DNA sequences from multiple loci. *Genetics*, 164(4): 1645–1656.

Shi, C. and Yang, Z. 2018. Coalescent-based analyses of genomic sequence data provide a robust resolution of phylogenetic relationships among major groups of gibbons. *Mol. Biol. Evol.*, 35: 159–179.

Yang, Z. 1994a. Estimating the pattern of nucleotide substitution. *J. Mol. Evol.*, 39(1): 105–11.

Yang, Z. 1994b. Maximum likelihood phylogenetic estimation from DNA sequences with variable rates over sites: approximate methods. *J. Mol. Evol.*, 39: 306–314.

Yang, Z. 2015. The BPP program for species tree estimation and species delimitation. *Curr. Zool.*, 61: 854–865.

Yang, Z. and Rannala, B. 2010. Bayesian species delimitation using multilocus sequence data. *Proc. Natl. Acad. Sci. USA*, 107: 9264–9269.

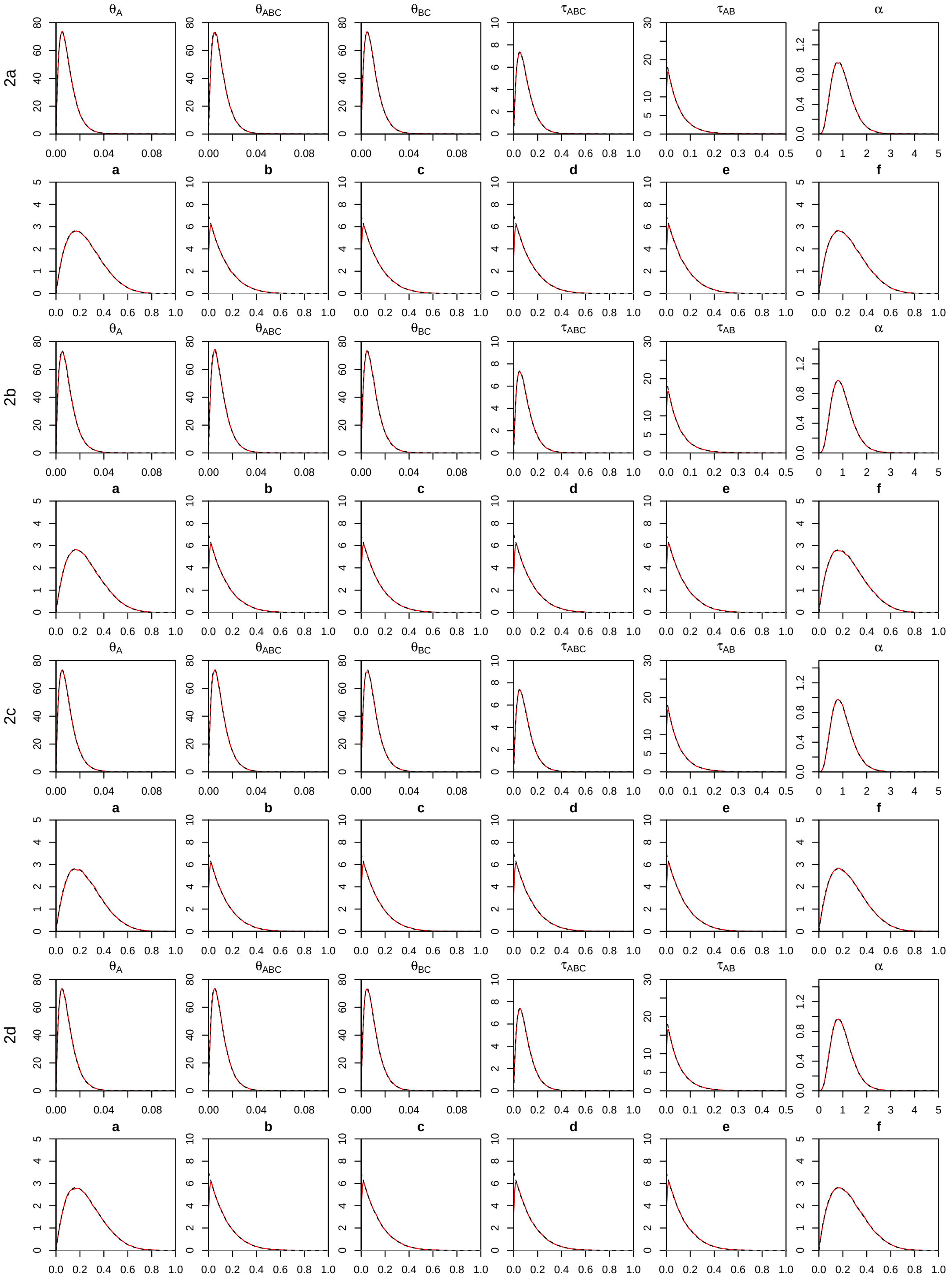

Figure S1: True (black dashed line) and empirically estimated (red solid line) prior distributions of 12 parameters under the independent-rates model (clock 2) when the likelihood function is fixed at 1. The true distributions are plotted using their analytical densities. The parameters include $\theta_A, \theta_{AB}, \theta_{ABC}, \tau_{ABC}, \tau_{AB}$ on the species tree (fig. 1a); these all have gamma distributions except τ_{AB}, the density of which is given in eq. A1. The other parameters are for locus 1. The gamma shape parameter for rate variation among sites α has a gamma prior, while the GTR exchangeability parameters (a,b,c,d,e,f) have marginal beta distributions beta$(2,6)$ and beta$(1,7)$. See SI text for details. The four relaxed-clock settings are

2a) `locusrate = 1 0 0 5 iid, clock = 2 10 100 5 iid LN` (i.i.d. prior for μ_i and ν_i among loci and log-normal kernel);

2b) `locusrate = 1 0 0 5 iid, clock = 2 10 100 5 iid G` (i.i.d. prior for μ_i and ν_i among loci and gamma kernel);

2c) `locusrate = 1 0 0 5 dir, clock = 2 10 100 5 dir LN` (`dir` prior for μ_i and ν_i among loci and log-normal kernel);

2d) `locusrate = 1 0 0 5 dir, clock = 2 10 100 5 dir G` (`dir` prior for μ_i and ν_i among loci and gamma kernel).

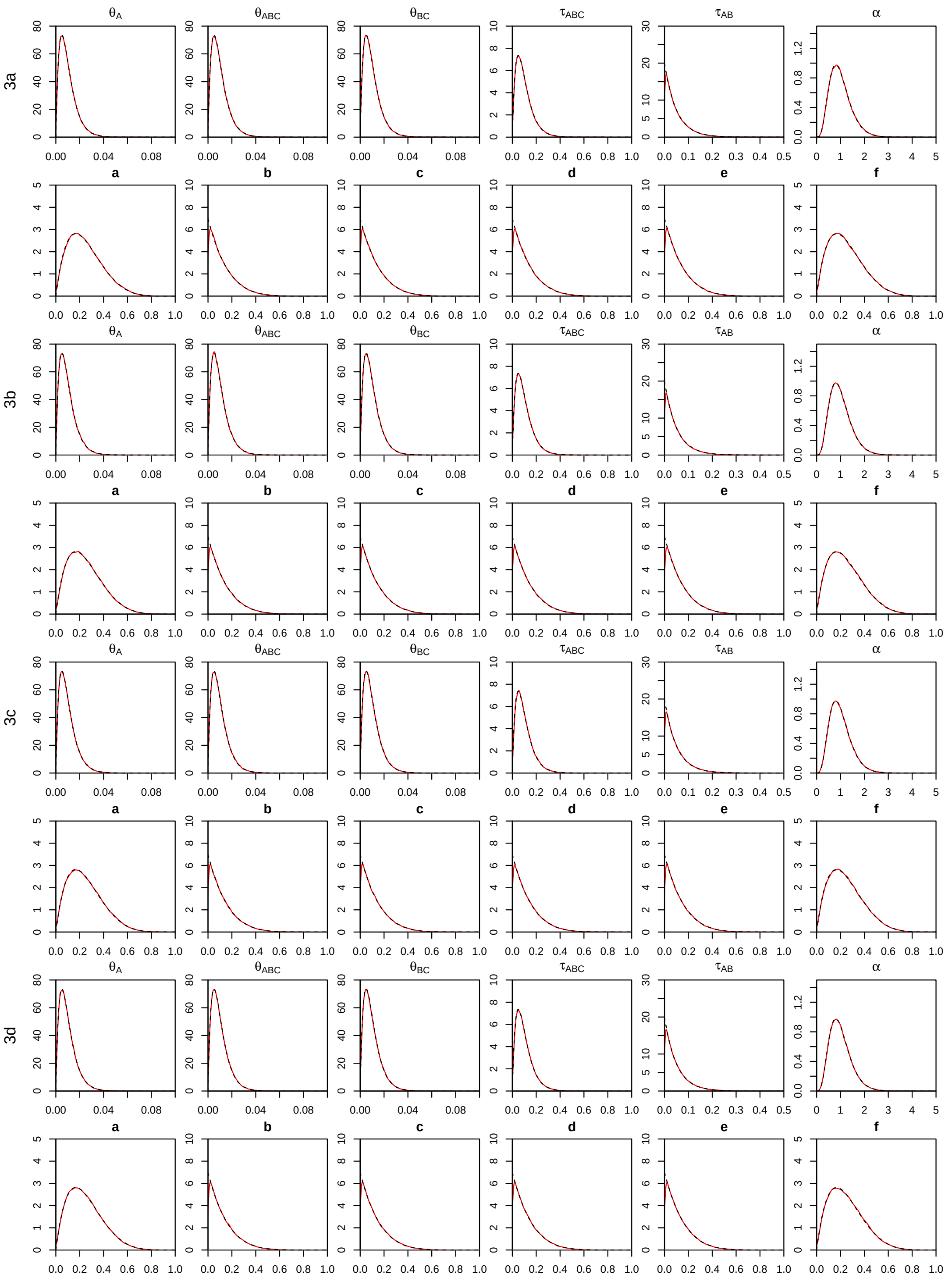

Figure S2: True (black dashed line) and empirically estimated (red solid line) prior distributions of 12 parameters under the correlated-rates model (clock 3) when the likelihood function is fixed at 1. See legend to figure S1 for definitions of parameters. The four relaxed-clock settings are
3a) `locusrate = 1 0 0 5 iid, clock = 3 10 100 5 iid LN` (i.i.d. prior for μ_i and ν_i among loci and log-normal kernel);
3b) `locusrate = 1 0 0 5 iid, clock = 3 10 100 5 iid G` (i.i.d. prior for μ_i and ν_i among loci and gamma kernel);
3c) `locusrate = 1 0 0 5 dir, clock = 3 10 100 5 dir LN` (`dir` prior for μ_i and ν_i among loci and log-normal kernel);
3d) `locusrate = 1 0 0 5 dir, clock = 3 10 100 5 dir G` (`dir` prior for μ_i and ν_i among loci and gamma kernel).

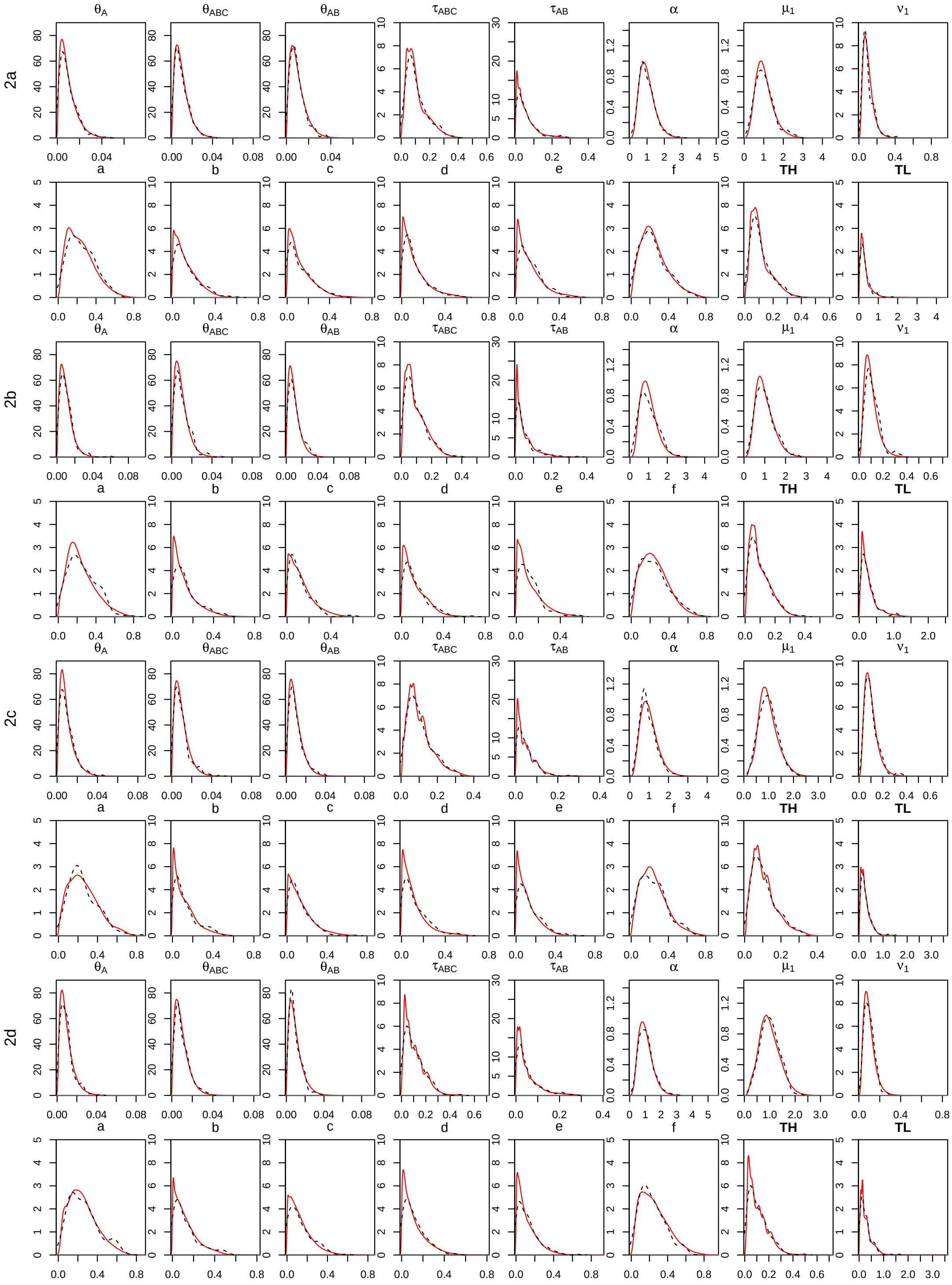

Figure S3: Prior (black dashed line) and average posterior (red solid line) distributions of 16 parameters or quantities in a Bayesian simulation under the independent-rates model (clock 2). TH is gene-tree tree height and TL is gene-tree tree length. The four relaxed-clock settings are:
2a) `locusrate = 1 0 0 5 iid, clock = 2 10 100 5 iid LN` (i.i.d. prior for μ_i and ν_i among loci and log-normal kernel);
2b) `locusrate = 1 0 0 5 iid, clock = 2 10 100 5 iid G` (i.i.d. prior for μ_i and ν_i among loci and gamma kernel);
2c) `locusrate = 1 0 0 5 dir, clock = 2 10 100 5 dir LN` (dir prior for μ_i and ν_i among loci and log-normal kernel);
2d) `locusrate = 1 0 0 5 dir, clock = 2 10 100 5 dir G` (dir prior for μ_i and ν_i among loci and gamma kernel).

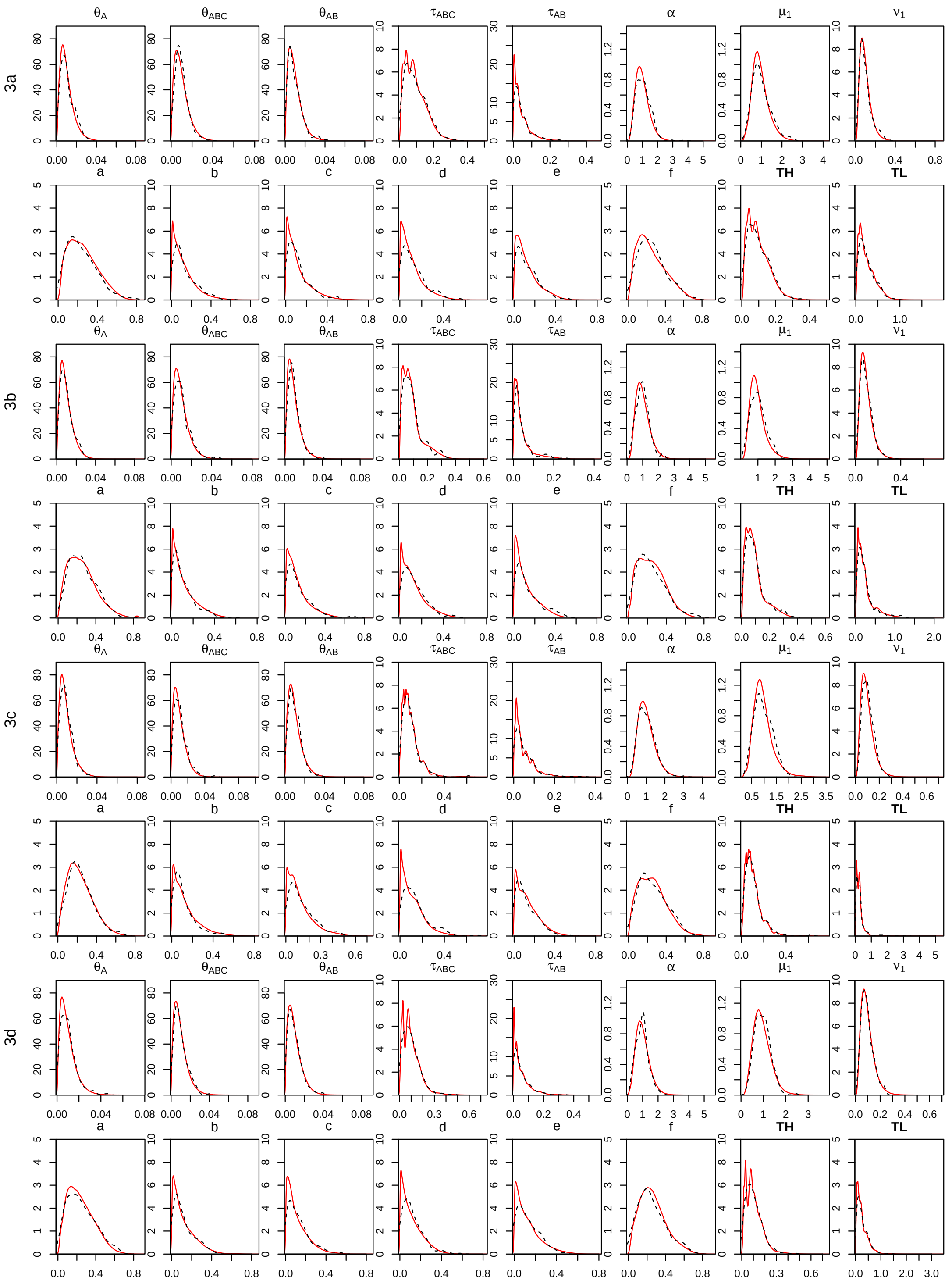

Figure S4: Prior (black dashed line) and average posterior (red solid line) distributions of 16 parameters or quantities in a Bayesian simulation under the correlated-rates model (clock 3) for four different settings:
3a) `locusrate = 1 0 0 5 iid, clock = 3 10 100 5 iid LN` (i.i.d. prior for μ_i and ν_i among loci and log-normal kernel);
3b) `locusrate = 1 0 0 5 iid, clock = 3 10 100 5 iid G` (i.i.d. prior for μ_i and ν_i among loci and gamma kernel);
3c) `locusrate = 1 0 0 5 dir, clock = 3 10 100 5 dir LN` (`dir` prior for μ_i and ν_i among loci and log-normal kernel);
3d) `locusrate = 1 0 0 5 dir, clock = 3 10 100 5 dir G` (`dir` prior for μ_i and ν_i among loci and gamma kernel).
See legend to figure S3.

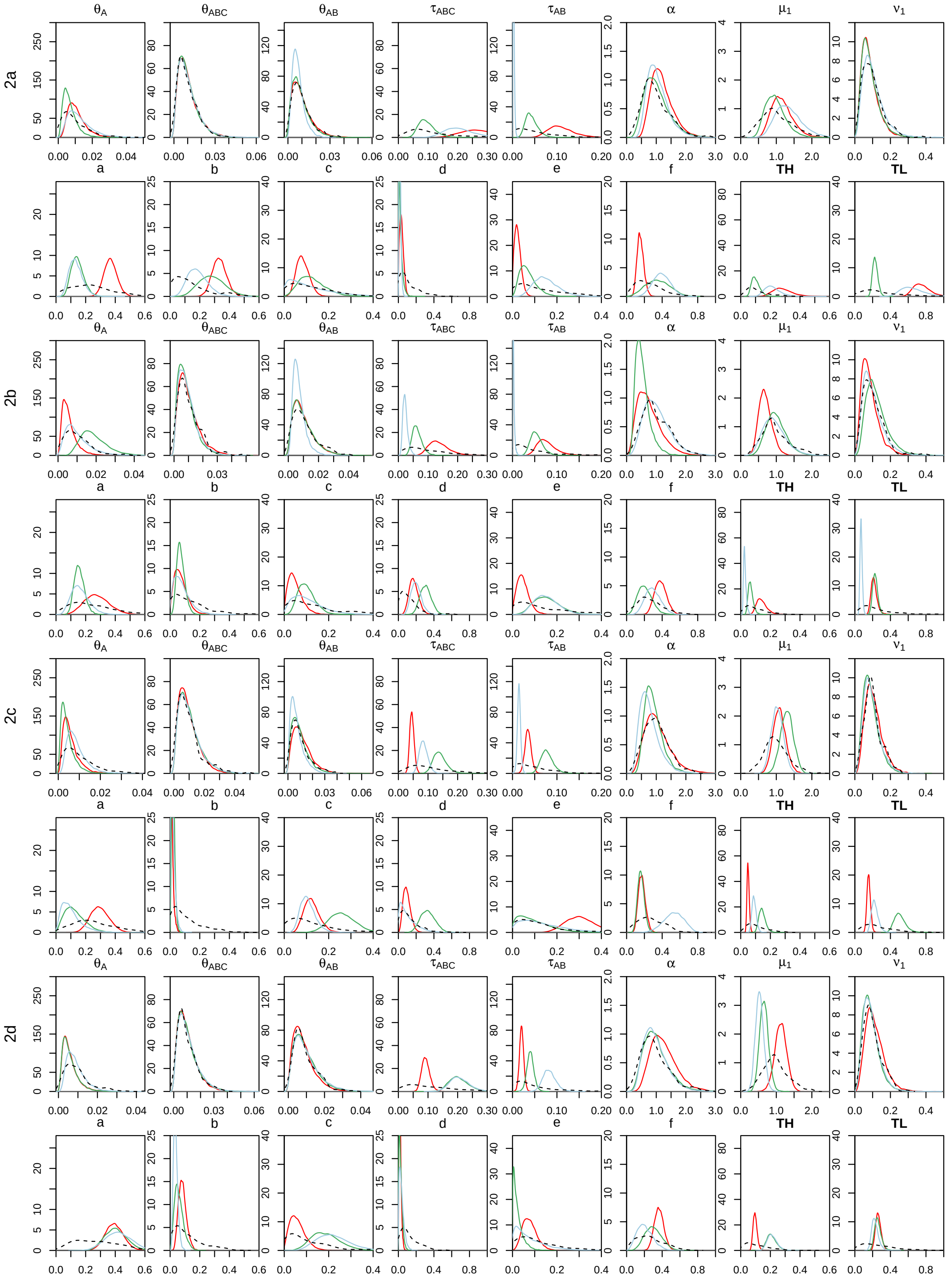

Figure S5: Posterior distributions for three replicate datasets in the Bayesian simulation of figure S3.

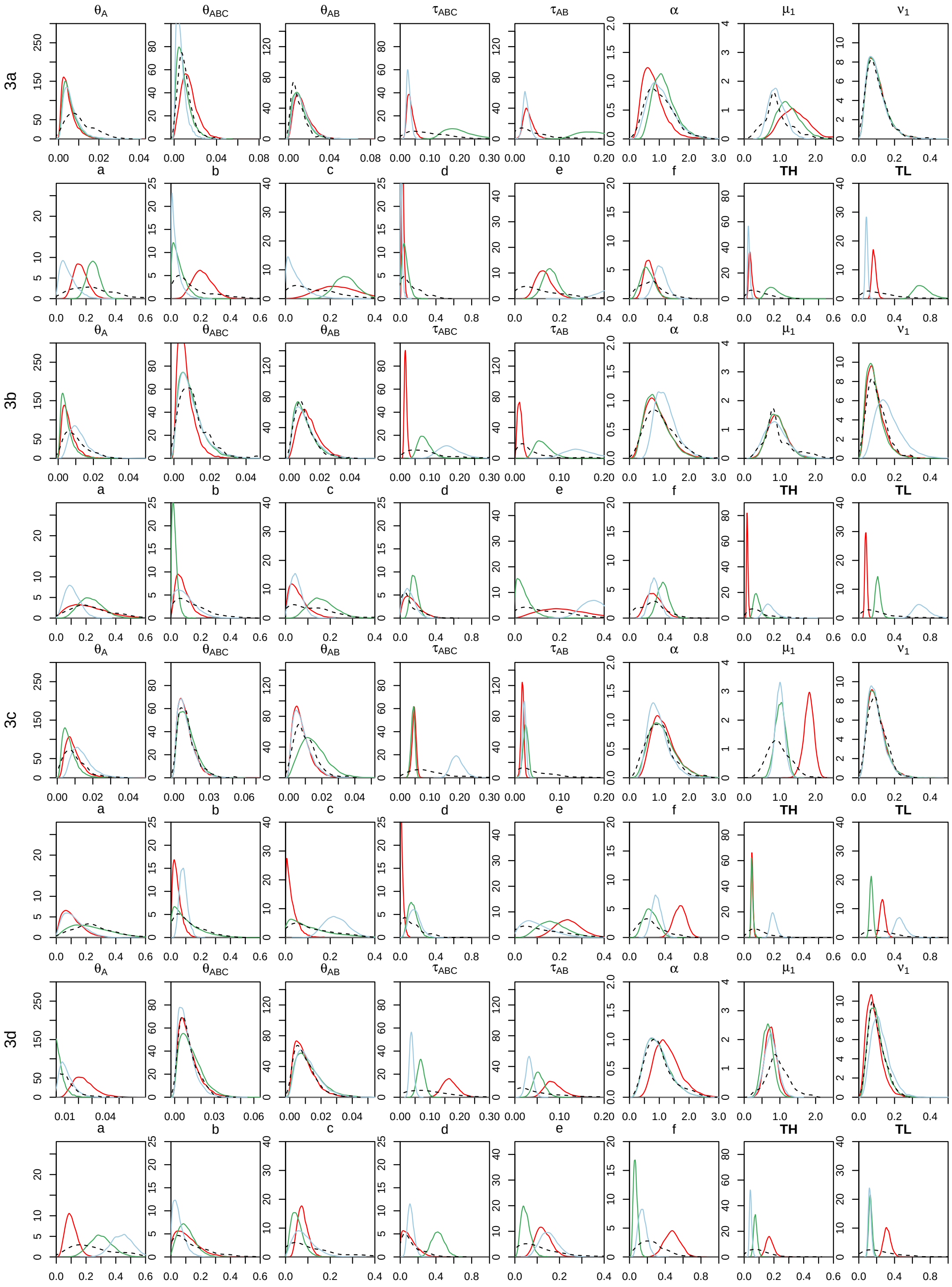

Figure S6: Posterior distributions for three replicate datasets in the Bayesian simulation of figure S4.

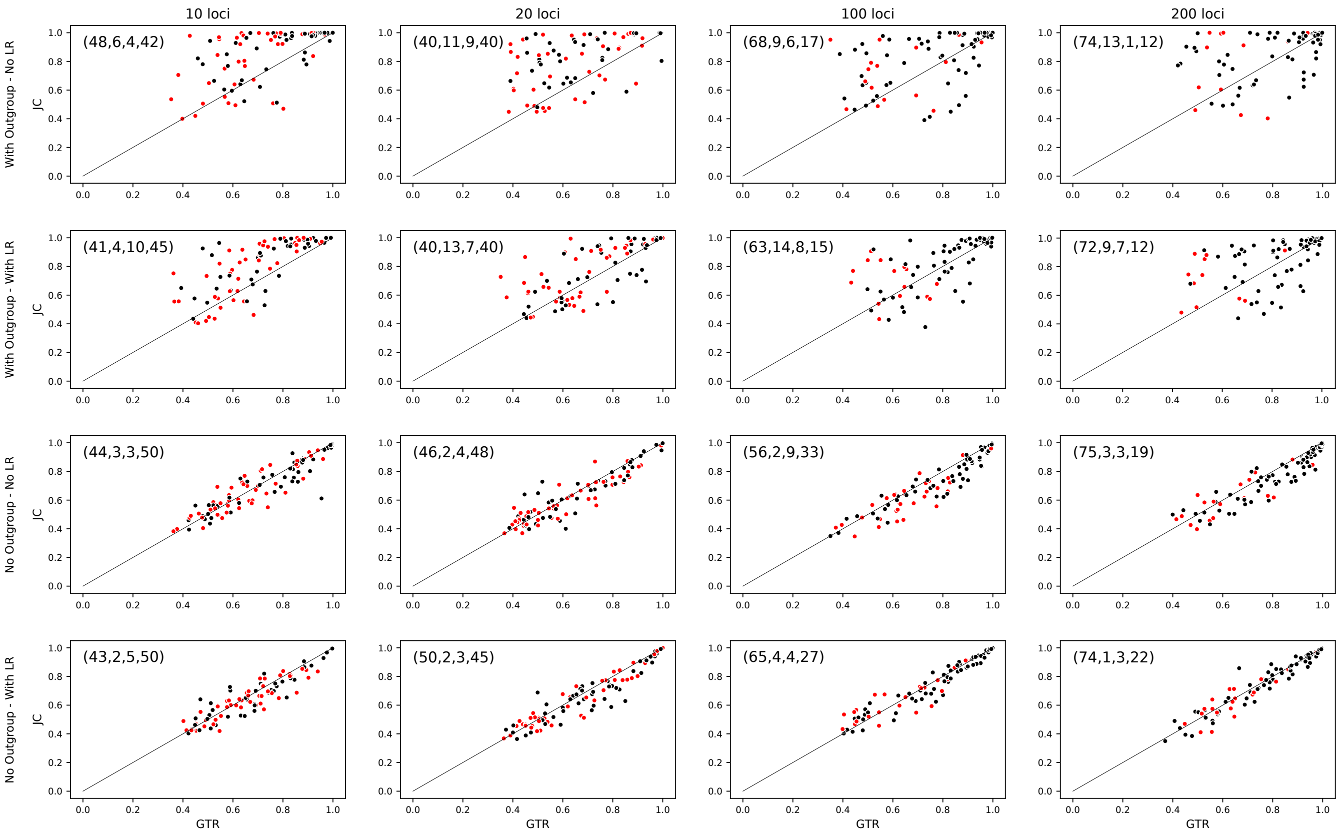

Figure S7: Posterior probabilities for the MAP tree inferred under the clock1+JC model plotted against those under clock1+GTR+Γ in BPP analysis of data simulated under GTR+Γ with $\bar{v} = 0.01$ (fig. 4 and table 2). Only replicates in which the two models inferred the same MAP tree are plotted, with black dots if the MAP tree is the correct tree and red dots otherwise. The first two rows are for data including an outgroup and the two last rows are without the outgroup. The model assumes either the presence of locus-rate variation (LR) or a constant rate for all loci (No LR). The counts in the upper left corner are in the order (TT, TF, FT, FF), with TT: GTR true tree/JC true tree, TF: GTR true tree/JC wrong tree, FT: GTR wrong tree/JC true tree, and FF: GTR wrong tree/JC wrong tree. For example, in the case of 10 loci with locus-rate variation, the counts “(48, 6, 4, 42)” mean that GTR+Γ was correct in $54 = 48 + 6$ replicates, while JC recovered the true tree in $52 = 48 + 4$ cases (table 2).

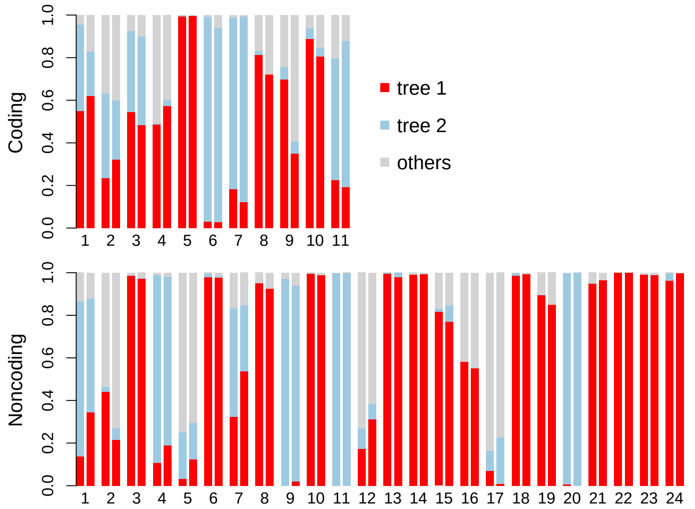

Figure S8: Posterior probabilities for species trees 1 and 2 (fig. 7) in the A01 analysis by BPP of blocks of loci from the gibbon genome under the JC and GTR+Γ_4 models. The strict molecular clock (clock 1) is assumed, with one rate for all loci, and the prior settings are the same as in figure 8. There are 11 coding blocks (each of 1000 loci) and 24 noncoding blocks (each of 500 loci), from figure 3A&B of Shi and Yang (2018), and each was analyzed under the JC (left) and GTR+Γ_4 (right) models. Data of block 1 were used in figures 8, 9, 10, & 11.

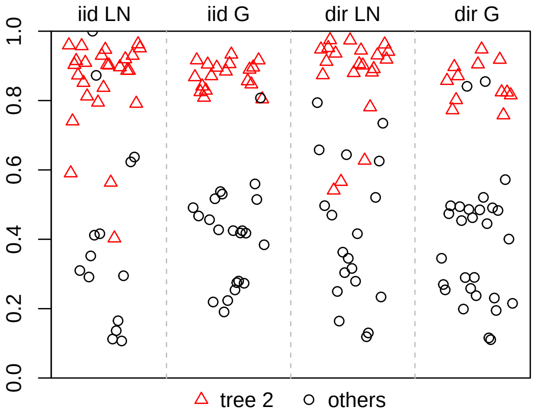

Figure S9: Posterior probabilities for the MAP tree in BPP analysis of the 250-loci ratite UCE data under four independent-rates models (clock), specified as
(a) `iid LN (locusrate = 1 0 0 5 iid, clock = 2 2 20 5 iid LN);`
(b) `iid G  (locusrate = 1 0 0 5 iid, clock = 2 2 20 5 iid G);`
(c) `dir LN (ocusrate = 1 0 0 5 dir, clock = 2 2 20 5 dir LN);`
(d) `dir G  (locusrate = 1 0 0 5 dir, clock = 2 2 20 5 dir G).`
For each of the four clock models, 40 replicate runs were conducted using four starting species trees and the differences among the replicate runs reflect MCMC mixing issues. Tree 2 (fig. 5) was the most common MAP tree.

(a) `MCcoal.ctl`

```
         seed = -1
      seqfile = mydata.txt
     Imapfile = Imap.txt
     treefile = mytree.tre
modelparafile = modelparas.txt

  species&tree = 4  A  B  C  O
                    1  1  1  1
  (((A #0.01, B #0.01):0.1 #0.05, C #0.01):0.105 #0.01, O #0.01):0.2 #0.01;

  loci&length = 200 500

   locusrate = 1 5 iid
       clock = 2 0.1 5 iid g

       model = 7
      Qrates = 0  10 5 5 5 5 10
   basefreqs = 0  10 10 10 10
alpha_siterate = 0  2  2  5
```

(b) `bpp.ctl`

```
           seed =  -1

        seqfile = mydata.txt
       Imapfile = Imap.txt
        outfile = out.txt
       mcmcfile = mcmc.txt
 constraintfile = outgroup.txt

     speciestree = 1 0
     speciesmodelprior = 1

     species&tree = 4  A  B  C  O
                       1  1  1  1
                    (((A, C), B), O);

        usedata = 1
          nloci = 200
      cleandata = 0
          model = gtr
     alphaprior = 2 2 5
     thetaprior = gamma 2 100
       tauprior = gamma 2 15
      locusrate = 1 0 0 5 iid   # (1: estimate locus rates mui, mubar=1 fixed)
*         clock = 1
          clock = 2 2 20 5 iid g
*         clock = 3 2 20 5 iid g

     finetune =  1: 0.01 0.001 0.02 0.001 0.001 0.001 0.0001 0.01

        print = 1 0 0 0 0  * mcmc sample, locusrate, heredity, gtrees
       burnin = 32000
     sampfreq = 2
      nsample = 200000
      threads = 2 1 1
```

Figure S10: (**a**) Sample control file `MCcoal.ctl` for simulating one replicate dataset of 200 loci (with sequence length of 500 sites) under the independent-rates model (clock 2). The example uses the GTR+Γ_5 model with $k = 5$ categories (`model` and `alpha_siterate`), sampling the base frequencies $(\pi_T, \pi_C, \pi_A, \pi_G)$, relative rates (exchangeabilities) (a,b,c,d,e,f) (Yang, 1994a) from Dirichlet distributions, and the gamma shape parameter from $G(2,2)$ (Yang, 1994b). (**b**) Sample control file `bpp.ctl` for species tree estimation (`A01: speciesdelimitation=0, speciestree=1`) using a dataset of 200 loci under the independent-rates model (clock 2) and GTR+Γ_5, with gamma priors on τ and θ.

Table S1. Species trees and node supports inferred by ASTRAL from three sets of ratite data (UCEs, Introns, and CNEEs) using different outgroups (ostrich and chicken, ostrich only, or chicken only)

	Ostrich and Chicken			Ostrich			Chicken		
No. loci	Tree	N1	N2	Tree	N1	N2	Tree	N1	N2
UCEs									
loci	1,997			2,054			2,278		
250	tree 1	0.82	0.48	tree 2	0.93	0.67	tree 1	0.53	1
500	tree a	0.54	0.33	tree 2	0.92	0.78	tree 1	0.67	1
1000	tree 2	0.50	0.80	tree 2	0.87	0.90	tree 1	0.95	1
All	tree 2	0.56	0.99	tree 3	0.62	0.98	tree 1	1	1
Introns									
loci	1,736			1,951			2,654		
250	tree 2	0.68	0.47	tree 2	0.95	0.43	tree a	0.44	1
500	tree 2	0.77	0.55	tree 2	0.90	0.69	tree a	0.51	1
1000	tree 2	0.86	0.53	tree 2	0.92	0.90	tree 1	1	0.86
All	tree 2	0.95	0.92	tree 2	1	1	tree 1	1	0.97
CNEEs									
loci	12,288			12,588			12,595		
250	tree 3	0.89	0.54	tree 3	0.97	0.55	tree 3	0.70	0.89
500	tree 3	0.69	0.85	tree 3	0.96	0.66	tree 1	0.77	0.86
1000	tree 3	0.48	0.99	tree 3	0.84	0.92	tree 1	0.88	0.98
2000	tree 1	0.62	1	tree 3	0.53	0.98	tree 1	1	1
4000	tree 1	0.81	1	tree 2	0.55	1	tree 1	1	1
8000	tree 1	0.92	1	tree 2	0.56	1	tree 1	1	1
All	tree 1	0.99	1	tree 3	0.45	1	tree 1	1	1

Note.— Trees 1, 2 and 3 are in figure 5, where nodes N1 and N2 are labelled. Tree a groups the rheas with the emu-cassowary clade (node N1), and then joins kiwis (N2). This tree is not supported in other datasets or analyses. Nodes other than N1 and N2 in the three trees of figure 5 had full support in all analyses and are not shown. The original datasets, including both ostrich and chicken as outgroups, had 3158 UCE loci, 5016 introns loci, and 12,767 CNEE loci (Cloutier *et al.*, 2019), whereas the total number of loci after our filtering is shown in the table. The subsets consist of the first sets of loci; for example, the 250-loci UCE dataset when the outgroup is ostrich consists of the first 250 of the 2054 loci.
